# Co-production of a consensus-based inclusivity checklist to promote inclusion and diversity in clinical research: a multi-method study

**DOI:** 10.1136/bmjopen-2026-127636

**Published:** 2026-10-27

**Authors:** Ann Cheng, Hardeep Aujla, Robert Grant, Chibuzo Mowete, Ali Alhashimi, Jeremy Dearling, Sarah Murray, Sue Page, Esther Mukuka, Gavin Murphy, Maria Pufulete, on behalf of the Equality, Diversity, and Inclusion Group of the National Cardiac Surgery Clinical Trials Programme, Enoch Akowuah

**Affiliations:** 1 Department of Cardiovascular Sciences, University of Leicester, Leicester, UK; 2 Queen Mary University of London, London, UK; 3 Sheffield Teaching Hospitals NHS Foundation Trust, Sheffield, UK; 4 NIHR, London, UK; 5 University of Bristol, Bristol, UK

**Keywords:** Research Design, Health Equity, STATISTICS & RESEARCH METHODS

## Abstract

**Abstract:**

**Objectives:**

To co-produce a consensus-based inclusivity checklist for widening inclusion of under-represented groups and standardising reporting of inclusivity activities in clinical research.

**Design:**

Formal consensus methodology including use of existing frameworks, scoping review, survey, community outreach and consensus development workshop, conducted in line with the EQUATOR (Enhancing the QUAlity and Transparency Of health Research) Network guidance for reporting guideline development.

**Setting:**

UK clinical research environment, including Clinical Trials Units (CTUs), academic institutions, healthcare settings and community groups.

**Participants:**

Patient and public contributors, including members of under-represented communities, researchers, clinicians, trial managers, CTU staff, funder and equality, diversity and inclusion experts.

**Methods:**

We updated a rapid scoping review of barriers and strategies to increase inclusion in adult clinical trials (MEDLINE, 2021–2024), undertook a national survey of UK Clinical Research Collaboration (UKCRC)-registered CTUs and conducted in-depth discussions with five under-represented community groups. Findings were synthesised using a conceptual framework spanning levels (participant, researcher, organisational, societal) and stages of the research lifecycle. A prototype checklist was developed and refined through iterative stakeholder review and finalised at a 1 day national consensus workshop using structured discussion.

**Results:**

We included 98 articles in the scoping review, identifying a broad but heterogeneous evidence base with limited high-quality evaluations of inclusion strategies. 37 respondents from 21 CTUs completed the survey (41% CTU response rate). The most effective inclusion strategies were perceived to be translation, videos or information in easy to read or visual formats and paying travel costs (32%, 30% and 24% of respondents, respectively). Most respondents stated that budgets were not enough to cover the activities required to recruit people from under-represented groups (64%). The final consensus-based checklist comprises 26 items across six domains: research priority setting, research inclusion planning, study design, delivery, data collection and reporting and dissemination and engagement. Most items were designated core, with optional items to allow flexibility across study contexts.

**Conclusions:**

We developed a practical, consensus-based inclusivity checklist that operationalises existing high-level guidance and supports researchers to embed inclusivity systematically throughout the research lifecycle. The checklist addresses a critical gap between principle and practice and provides a foundation for improving transparency, consistency and equity in clinical research participation.

**STRENGTHS AND LIMITATIONS OF THIS STUDY**

- The inclusivity checklist was developed using robust methods, involving iterative refinement and multi-stakeholder consensus.
- The co-production process involved researchers, clinicians, funders, equality, diversity and inclusion (EDI) and patient and public involvement (PPI) experts and community members, highlighting the multiple perspectives and practical realities around inclusivity in research.
- Under-served communities and PPI were at the core of the development process and enabled meaningful co-production of the inclusivity checklist.
- Community outreach was limited to a small number of groups in the local area due to logistical and financial constraints, which may limit the breadth of perspectives represented.
- The acceptability, feasibility and impact of the inclusivity checklist are yet to be evaluated.

## Introduction

 Women, ethnic minorities, people with lower socioeconomic status, older and younger people and those with disabilities are examples of groups that are consistently under-represented in clinical studies.^1 2^ Barriers such as access and opportunity, geographic location, language, cultural differences and logistical constraints further compound under-representation. Collectively, this under-representation leads to evidence gaps and reduced generalisability of findings.^3–6^

The inclusion of under-represented groups in clinical research has become a central priority for funding bodies, regulators and patient communities worldwide. Several guidance documents and policy frameworks already seek to improve inclusion in research, including the National Institutes of Health requirements on sex and race reporting, the UK National Institute for Health and Care Research (NIHR) INCLUDE framework, Strategies for Trialists to promote Equal Participation in clinical trials for Under-served Populations and REP-EQUITY: a toolkit for capturing a REPresentative and EQUITable sample in health research.^7–10^ Internationally, legal requirements such as the Pediatric Research Equity Act (PREA) and the mandated Diversity Action Plan by the Food and Drug Omnibus Reform Act of 2022 have further highlighted the importance of research inclusion.^11–13^ These frameworks provide high-level principles and, in some cases, population-specific considerations (eg, ethnicity), but they offer limited operational detail on how research teams should systematically embed inclusivity across the entire research pathway. Because they lack sufficient specificity to drive behavioural change in day-to-day research planning, delivery and reporting, their implementation remains variable in practice and trial teams frequently report uncertainty about which concrete actions to take and at what stage.^14^

There is evidence that guidance provided in the form of an operational checklist that tells researchers what practical steps to take can standardise practice and ensure key elements of the guidance are not overlooked.^15 16^ Consensus-based checklists have been successfully developed in other areas of clinical research, such as Consolidated Standards of Reporting Trials (CONSORT) and Preferred Reporting Items for Systematic Reviews and Meta-Analyses (PRISMA). These have demonstrably improved transparency, rigour and reporting quality.^17 18^ A similar checklist specifically on inclusivity could play a critical role in bridging the gap between principle and practice, by providing a structured mechanism for researchers to embed inclusivity considerations across all stages of the research pathway—from protocol development to recruitment, data collection and dissemination.

The objective of this study was therefore to co-produce a consensus-based inclusivity checklist for clinical research including clinical trials and prospective observational studies. The checklist is intended to serve as a practical tool for researchers to consider and document inclusivity throughout the research lifecycle, adapting flexibly to the study context rather than as a set of mandatory requirements. It can also be used at the reporting stage to document the inclusivity activities undertaken and identify areas where these were not possible.

## Methods

We developed the checklist using the methodological framework for developing reporting guidelines suggested by the EQUATOR Network.^19^ We used formal consensus methods (evidence from the published literature and expert opinion) to develop and refine checklist domains.

### Conceptual framework

We conceptualised inclusivity as being shaped by factors at multiple levels (participant, researcher, organisational and societal) and across key stages of the research lifecycle (from initial concept and funding application through protocol development, recruitment and retention, data collection and analysis, and dissemination). Our working premise was that an operational checklist could support behaviour change by prompting research teams to anticipate common barriers and plan practical, evidence-informed strategies at each stage. Accordingly, the checklist was designed to align specific actions with both stages of the research pathway and the level at which barriers primarily arise.

### Formal consensus working group and steering group

We assembled a working group comprising two clinicians (AC and RG), one methodologist (MP) and one trial manager (HA), responsible for conducting the scoping review, survey and community outreach, assembling the first draft of the checklist and revising it through multiple iterations. The Working Group managed the study, collated and presented the evidence, and met twice weekly to discuss progress and allocate tasks.

We also assembled a UK-wide steering group of 40 members including patient and public representatives (5), patient and public involvement (PPI) engagement officers (2), academic (multidisciplinary) clinicians (19), researchers (4), research delivery staff from Clinical Trials Units (CTU) (3), and individuals with roles in Equality, Diversity and Inclusion (EDI) in research (1) representing a diversity of views and experience. Membership to the steering group was advertised nationally in the UK through the National Cardiac Surgery Clinical Trials Programme, Clinical Trial Units and word of mouth invitation; membership was open to all interested individuals. The steering group met bi-monthly between Feb 2024 and March 2025 to review the evidence on which the checklist was based and each iteration of the checklist.

### Scoping review

We updated the rapid review by Bodicoat *et al*.^20^ We registered the protocol (https://osf.io/28xdp) and informed the authors of the planned update. The search strategy (online supplemental table S1) was revised with their input and utilised the concepts of “underserved”, “barriers”, “facilitators”, “inclusion” and “clinical trials” using keyword searches. We searched one database (Medline Ovid), with publication date from 1 March 2021 to 22 March 2024, in line with their rapid scoping review approach. Inclusion criteria were: study reported in the English language using any design that addressed/discussed barriers to inclusion or strategies to improve inclusion in clinical trials recruiting adults (≥18 years old). We excluded articles that used inequality characteristics to adjust for prognosis or report results or studies that described inequitable access to care. For example, studies which adjusted their result by subgroups such as age or sex without outlining strategies for research inclusion were excluded. We also excluded letters, editorials and studies in children. Although commentaries were initially excluded, a subsequent decision was made to include them as some reflected on the effectiveness of strategies implemented in trials to improve inclusion.

We used a rapid scoping review approach and did not search multiple databases or include studies published in languages other than English, as the purpose of the review was to inform a live consensus process within a limited timeframe. This was also justified by our prior knowledge that the evidence base is limited, which underpinned the use of consensus methods, and the expectation that barriers and facilitators to inclusion, and strategies to increase inclusion, are likely to be finite.

Screening by title and abstract was performed by one reviewer, with a second reviewer screening a 10% random sample to ensure consistency; discrepancies were resolved through discussion. We developed a data extraction form (online supplemental table S2) and extracted the following data from eligible full-text articles: first author; journal; year; country; study design; number of participants (if applicable); under-represented characteristics; and barriers and/or facilitators of research inclusion. Data extraction was performed by four reviewers with an independent reviewer extracting data on 10% of eligible full-text studies to ensure agreement. We used ‘saturation’ as a criterion for discontinuing data extraction, defined as review of full text of 10 consecutive studies without identifying any new barriers or strategies.^21^ Studies were selected randomly from all eligible full-text studies and data extraction continued until the saturation criterion was met. We did not perform risk of bias assessment given that the aim was to map barriers and strategies and that the main output was descriptive. We included all study designs, and we did not extract quantitative data (eg, numbers of patients with under-represented characteristics recruited). Data were iteratively categorised into our conceptual framework, resulting in the following final themes: trial design, communication, recruitment and retention and participant- and researcher-related factors.

### Survey

We undertook a national survey of UK Clinical Research Collaboration Registered CTU to identify barriers and strategies that researchers currently use and believe are successful to increasing recruitment of under-represented groups in order to prioritise and refine the checklist content. We developed a questionnaire including 20 questions: CTU and respondent characteristics (6); strategies employed in the CTU for increasing inclusivity in trials (unit wide and trial specific) and barriers to implementation of these strategies (13); PPI in trials (1). Item content was informed by the findings of our scoping review, existing guidance^7–10^ and discussion with the steering group. The draft questionnaire underwent six iterations. It was piloted with steering group members and two staff from selected CTUs to assess clarity, relevance and completion time; minor wording changes were made in response to feedback. The questionnaire (online supplemental table S3) was administered using Joint Information Systems Committee Online Surveys. The link was emailed to each CTU between September and October 2024, with a reminder email for non-completion. CTU could forward the survey to any relevant staff to complete. There was no limit to how many responses each CTU could submit, and no compensation was provided for completion.

### Community outreach

Between February 2024 and December 2025, we conducted in-depth discussions about perceived barriers to participating in research and potential solutions with five community groups (Leicester, UK) representing diverse characteristics (between 2–6 face-to-face meetings with each group). These included Asian British women, elderly people from mixed backgrounds, people with lower English proficiency or lower socioeconomic status and an African-Caribbean church group (online supplemental table S5). These groups were chosen based on pre-existing partnerships established by the NIHR Leicester Biomedical Research Centre’s Patient and Public Involvement team, as well as partnership from other external research projects. While these groups are all based in Leicester, UK due to logistical and resource challenges for the working group (AC, HA, RG), Leicester is well-placed as one of the most diverse cities in the UK, a ‘minority majorities’ city where more than 50% of the population are of non-white ethnicity. The variation in the number of meetings with each group was due to a combination of logistical arrangements as well as the time allocated for each session (ranging from 30 min to 2 hours). Compensation in the form of free dance classes, provision of physical activities and games and lunch were provided accordingly.

Themes used to guide the discussions were informed by the findings of the scoping review and existing guidance^7–10^ and were organised as follows: (1) an introduction to research, highlighting the disparity in equality and inclusion; (2) personal experience with research; (3) exploration of barriers, highlighting the contextual factors and needs of the different groups; (4) discussion of known facilitators and preferences by community (eg, inclusion of family member who can translate is preferred over a translator), (5) accessibility of clinical trials. Detailed notes of the discussions were taken by at least one member of the working group and summarised into key barriers, facilitators and suggested strategies, which were then organised into preliminary themes aligned with those from the scoping review and survey.

### Draft checklist development

The first prototype of the checklist was devised based on our conceptual framework (participant, researcher, organisational and societal levels) and mapping the barriers and strategies for addressing the barriers against one another using data from the scoping review, survey, community outreach and existing inclusion frameworks (online supplemental table S6). These were mapped onto processes of the research cycle (eg, protocol development, recruitment and retention, dissemination) by the Working Group based on their expertise and knowledge about inclusivity and research processes. For example, lack of transport to a research centre (participant barrier) was matched with provision of travel arrangement (facilitator) and grouped with other logistical factors mapping to research recruitment.

The prototype was iteratively refined by the steering group and community outreach groups, who reviewed and commented on the developing prototype (email communication and online meetings for the steering group and face-to-face meetings with community outreach groups) and made decisions about what to include and exclude from the checklist informed by the available evidence.

### Consensus workshop

The final checklist prototype was finalised in a 1-day consensus workshop (Leicester, December 2024). The aim was to identify any strategies missing from the checklist, revise the text in the checklist to improve clarity, identify further examples of good practice and establish the minimum set of strategies which should apply to all studies. In order to balance continuity, stakeholder expertise and new independent perspectives, invitations to attend the workshop were sent out via the National Cardiac Surgery Clinical Trials Programme, community outreach, and word-of-mouth invitation, with an emphasis for intended users of the checklist in the research setting as well as patient and public partners to ensure co-production. Attendees external to the steering group were prioritised to offer potentially alternative perspectives to the draft checklist.

The workshop was attended by 38 participants from across the UK, representing all stakeholders involved in research, including: PPI (20, of which 8 were from community outreach); research nurses (2); researchers (3); academic healthcare professionals (9), project manager (1), funder representative (1), EDI expert (1); administrator (1); members of the working group (4); members of the steering group (4). The workshop was structured as follows: short presentations of the evidence on barriers and strategies gathered from the scoping review, survey and community outreach, followed by three breakout sessions to: (1) review checklist; (2) identify case studies and examples of good practice; (3) define minimum criteria for researchers to apply when using the checklist (online supplemental table S8).

The day followed a consensus development conference approach; no formal voting or majority-rules process was used. Attendees were split into four groups according to expertise (with PPI members evenly distributed between groups) to discuss 1–3 above for specific domains on the checklist (study design, reporting and dissemination; study delivery including recruitment and retention; research inclusion plan; role of community advisory groups and partnerships). Each group had two facilitators, one of which was a PPI member. After each breakout session, the whole group convened for feedback and discussion. During the whole group discussion, items in the checklist were reworded, combined or deleted (if attendees felt they were repetitive or redundant), with consensus reached through facilitated discussion and agreement.

### Patient and public involvement

The development of the checklist had PPI embedded at every stage of the research as described in the methods.

## Results

### Scoping review

A total of 2495 articles were identified for screening (after duplicate articles were removed), and 98 full-text articles were included when the saturation criterion was met (figure 1). Internal discrepancy occurred in 4.6% (49/1055) of the double screened articles and 5.1% (5/98) of the data extraction process; these were all resolved through reviewer discussions. Final articles included: reviews (42, of which 13 were systematic reviews), reports and evaluations of recruitment methods in non-randomised studies and randomised controlled trials (16), mixed methods studies (6), questionnaires (5), qualitative studies (12), commentaries (12) and other (5).

**Figure 1:**
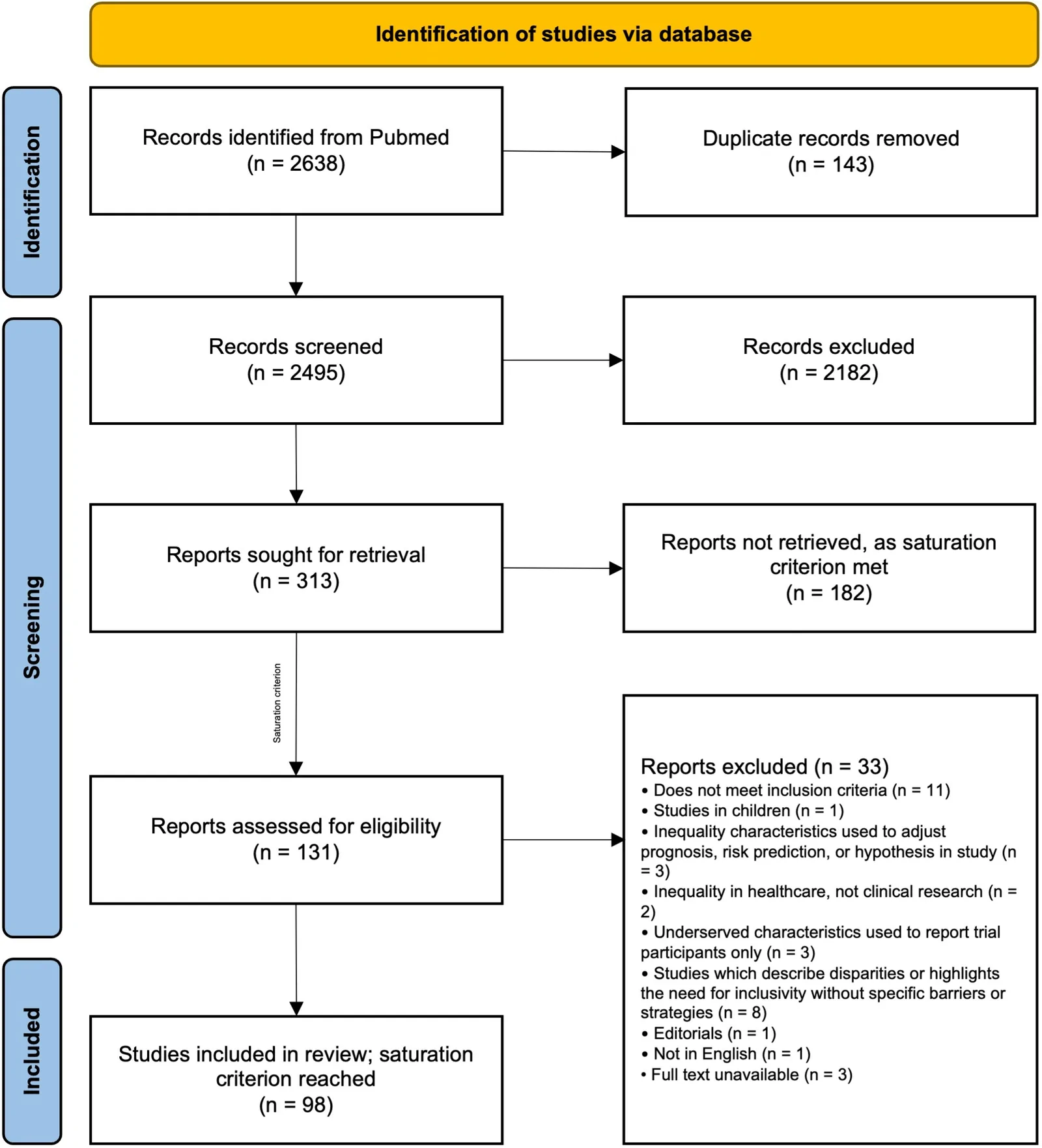
PRISMA flow diagram. PRISMA, Preferred Reporting Items for Systematic Reviews and Meta-Analyses.

Table 1 presents a summary of the facilitators identified by both Bodicoat *et al* and our scoping review, categorised into themes according to our conceptual framework. Strategies which could be considered under multiple themes were listed under one theme for pragmatic purposes; for example, use of multimedia material in recruitment material could be considered under both ‘recruitment and retention’ or ‘communication’ but we included it under communication for the purposes of analysis.

**Table 1:** Recommendations for improving inclusion by Bodicoat *et al* and additional strategies identified from scoping review

	Bodicoat *et al*^20^	Present review update, 2024 highlighting additional strategies
Trial design	Establish a diverse community advisory panel for ongoing input into the research process	Develop a diversity plan within the trial and appoint a diversity officer Consult with dedicated service on enhancing community engagement and research inclusion Create a flexible study protocol to address barriers to participation
Recruitment and retention	Include education about clinical trials for the public on study websites Encourage the use of sites with high enrolment of under-served groups Consider creating a local registry of individuals from under-served groups who are interested in participating in trials Explore whether linkages with non-healthcare data sources could increase trial access for people without access to healthcare Address logistical barriers to participating in research	Design sub-studies to target minority populations Consider ‘enrichment’ if not meeting recruitment targets for under-represented groups use innovative research delivery such as decentralised trials and hub-and-spoke delivery
Communication	Personalise communications as far as possible Have video calling as a contact option Alternative language options available for all communications Consider offering community outreach Include social proof where possible in communications Offer extended office hours	Incorporate visual and text-enhancing images in study materials Align study material with participant motivation (eg, altruism, emotional appeal) Develop accessible study material, such as the use of health literacy guidelines, cultural adaptation of material, and use of images reflecting population of interest Use branding and marketing strategies to promote study
Participant factors	Examine demographic summary of excluded populations Collect data on both sex at birth and gender identity	(No new themes identified)
Researcher factors	All research staff should receive cultural competency training Increase recruitment of staff from under-served groups	Increase staff capacity and training (eg, time required to build rapport with participants and additional training such as implicit bias and inclusive communication methods)

### CTU survey

The survey was shared with 51 UKCRC registered CTUs and was completed by 37 respondents across 21 of these CTUs (41% CTU response rate), with the majority of respondents in a trial management position (45%). Only around one third of respondents reported that their unit had a designated EDI lead (30%) or a unit-wide EDI strategy (32%, existing or being developed). EDI strategies were mostly considered in recently funded trials or trials in set-up. Most of these strategies were based on existing guidance, with the NIHR-INCLUDE Guidelines being the most popular, used by 43% of respondents. Most strategies were focused on the planning (73% of respondents), preparatory, for example, protocol/patient materials drafting (76% of respondents) and setup (68% of respondents) stages of trials. The under-represented characteristics most commonly considered were ethnic diversity, socio-economic disadvantage and language barriers (65%, 57% and 51% of respondents, respectively).

Approaches to barriers and strategies varied considerably across CTUs and different contexts. The most commonly reported barriers to recruiting under-represented groups were communication (65%), participant-level barriers, for example, mistrust, fear (46%), insufficient funding (43%), accessibility (41%) and trial design (38%). The most effective perceived strategies were translation of participant-facing materials (68%), travel costs (65%), accessible recruitment materials (65%), incentive payments (54%) and interpreting services (49%). 64% of respondents reported that EDI budgets probably or definitely did not adequately cover EDI activities. The complete survey responses are available in the online supplemental table S4.

Free text responses further highlighted a ‘lack of robust data to identify which groups are not being recruited’, that there is currently ‘no clear evidence’ on the benefits of EDI strategies on actual research inclusion, and that ‘strategies have added very little’ or ‘nil’, and that ‘EDI in healthcare is a bigger issue than just trials; once we improve general healthcare engagement this should improve trials EDI’.

### Community outreach

Between February and December 2024, we undertook 13 discussion sessions with the different community groups. The key findings from the discussions are briefly summarised in table 2 below (also mapped in online supplemental table S6).

**Table 2:** Key findings from community outreach discussion

Discussion themes	Key findings
Introduction to research	Opinions of research are generally positive, often citing altruistic motivations. However, using Covid vaccine trials as an example, the majority would choose not to participate in research due to fear (of unknown risks) and ‘not wanting to be the guinea pig’, ie, exploitation
Personal experience	Mixed positive and negative experience in participants who have previously joined research studies: Lack of result dissemination (‘don’t know what happened with the study’ and didn’t know who to contact) led to a sense of ‘overpromise’ when participants didn’t get anything in return. One participant felt disappointed as it was an investment of their time and interest Positive experiences from research studies as educational opportunities to learn more about their own medical conditions and having access to clinical care above what would ordinarily be available
Exploration of barriers	Barriers to participation are manifold, including: Language barriers Logistical barriers (especially parking) Understanding of research (including risks or invasiveness of procedures were concerns) Relevance of study to participant Who is recruiting Other personal commitments (eg, family or care responsibilities) Anxiety and lack of confidence, especially for women in a male-dominated culture Digital and technology requirements Duration of study visits Poor communication Data privacy concerns, fears of fake requests to join research Trust issues, stigma Cultural reasons where people do not feel comfortable talking about health issues without their family Feeling of being ‘written off’ due to not meeting eligibility criteria
Known facilitators and preferences by community	Language barriers can be addressed by dedicated research staff who speak the same language and more so by inclusion of family members Free parking, free transport, General Practitioner as a trusted source, testimonial by friends and family, shorter and clearer information around inclusion criteria, no jargon, financial incentives and compensation, aligning research and clinical visits, home visits, face-to-face interaction over remote visits, regular follow-up, written information for participants to refer back to, choice of communication preferences (eg, telephone, video, in-person) Use of ‘advocate’ who is a member of the community and involved in the research design Preference for intervention being evaluated in trial Research in a group setting Rapport building between researcher and participant through open, honest and transparent conversations
Accessibility of clinical trials	Lack of awareness and access to active research limits participation. Additional accessibility requirements (eg, internet, being able to text) further limit participation

### Checklist development

We initially mapped all the barriers and strategies identified from the scoping review, survey, community outreach and expert opinion from the steering group into defined themes based on our conceptual framework, indicating the evidence source for each (online supplemental table S6). The core working group developed these themes into 57 statements, incorporating text to describe how to achieve each strategy. This formed the first checklist prototype, which was iteratively refined (through five cycles) through meetings with the steering group and community groups, reducing the number of statements from 57 to 40 (online supplemental table S7).

The final prototype was refined at the consensus workshop in December 2024. The consensus group reduced the number of statements from 40 to 26, by combining similar items and removing redundant items, and re-worded checklist items to improve clarity. No minority or dissenting opinion was identified. The final checklist of 26 items (table 3) is organised into six domains: (1) setting research priority and research question (four items); (2) research inclusion considerations (five items); (3) study design (five items); (4) study delivery (nine items); (5) data collection and reporting (two items); and (6) impact, dissemination and engagement (one item). Items 1–8, 10–12 and 15–26 were designated as core items that should be considered for all clinical studies, whereas items 9, 13 and 14 were considered optional, recognising that they may not be possible or necessary in every context (eg, where community advisory groups are not available). Each item includes a brief description and, where relevant, an example of good practice to illustrate how it can be implemented. The checklist was finalised with the addition of a glossary of terms, appendices and case studies. Full guidance accompanying the checklist can be found at: https://le.ac.uk/cardiovascular-sciences/about/heart-surgery/inclusivity-checklist (also online supplemental table S10). Users are encouraged to apply the checklist prospectively during study planning and protocol development, and to revisit it at key milestones (eg, site set-up, recruitment, analysis planning).

**Table 3:** The inclusivity checklist

	Checklist item	Yes	No	NA
**Setting research priority and research question**				
1	Describe the diversity of the target population.			
2	Engage with under-represented communities relevant to the target condition.			
3	Include members of the community as research partners.			
4	Evidence activities that raise awareness of and promote interest in the study among under-represented communities.			
**Research inclusion considerations**				
5	Describe the research inclusion plan in the study protocol.			
6	Budget for the research inclusion plan in the funding application.			
7	Designate individual(s) responsible for delivering the research inclusion plan.			
8	Document diversity of the research team.			
9	Embed an evaluation of the research inclusion plan in the study. (*optional*)			
**Study design**				
10	Describe how inclusion and exclusion criteria mitigate discriminatory bias.			
11	Document how protocol flexibility accommodates the needs of under-represented groups.			
12	Pre-specify representative recruitment targets, ensuring these are reviewed throughout the research.			
13	Document the use of decentralised trial methods (move research delivery into communities) or non-traditional trial designs. (*optional*)			
14	Include options for partial participation, assent, and permission for future study contact in the consent form. (*optional*)			
**Study delivery**				
Recruitment and retention strategies				
15	Document how recruitment strategies are multiple, diverse and flexible to address inclusivity.			
16	Specify mechanisms of feedback about recruitment and retention with recruiting sites and under-represented groups.			
17	Document plan for inclusion of research sites from geographically under-represented areas.			
18	Document that research staff have received training in cultural competence, implicit bias and inclusive communication.			
19	Document how people with lived experience of the target condition, clinicians and healthcare professionals have helped with recruitment of under-represented groups.			
20	Document strategies used to increase accessibility for participants from under-represented communities (eg, compensation, logistics and travel arrangements).			
Communication strategy				
21	Document steps to ensure study materials such as patient information leaflets and consent forms are short, simple and written in easy-to-read language.			
22	Document the strategies available throughout all stages of the research for people with communication difficulties (eg, do not speak English, sight or hearing impairments).			
23	Document how communication strategies reflect participant preference.			
**Data collection and reporting**				
24	Collect comprehensive demographic data on participants.			
25	Report results stratified by under-represented characteristics or sub-groups.			
**Impact, dissemination and engagement**				
26	Document dissemination of research results to participants and wider relevant communities.			

## Discussion

We developed a consensus-based inclusivity checklist for clinical research, aimed at supporting researchers to systematically embed inclusivity across all stages of the research pathway, from study planning and design through recruitment, delivery, data collection and dissemination. The checklist provides practical, operational guidance and encourages documentation of all inclusivity activities. It is not intended to function solely as a reporting checklist; instead, it is flexible for researchers to apply at any stage of their study. This may include the design of a study, ensuring adequate plan and costing for inclusivity prospectively; mid-course, allowing researchers to incrementally enhance inclusivity even in ongoing studies; or reporting, documenting inclusive activities or identifying areas where these were not possible. The checklist should be adapted to the study context rather than treated as a mandatory set of requirements, assessing the extent to which the embedded inclusion strategies have been successful. It can also be used by patient partners, funders and, potentially in the future, journals to gauge how far inclusivity has been embedded in the research.

We used robust methodology to develop the checklist, including scoping review, CTU survey, community engagement and expert opinion. Engagement with under-represented communities during the development process helped us refine the tool to ensure that strategies were acceptable, relevant and culturally appropriate. The scoping review highlighted that a large body of literature on research inclusion exists and is increasing exponentially, but also that there are few high-quality studies that provide definitive evidence for the use of any strategy in the checklist. This was confirmed in the survey of CTUs, where respondents felt that there is a lack of robust data showing which strategies work and for whom.

High-quality evidence on what works and for whom could be generated through studies within trials (SWATs) that embed and test specific inclusivity strategies in recruiting trials. However, there are few SWATs addressing inclusion/diversity in trials and many focus on increasing overall recruitment rather than any specific under-represented group.^22–24^ Recently, the York Trial Forge SWAT Centre and SWAT Network has produced a list of 11 priority recruitment and retention SWATs, with one priority question addressing the most effective strategies to recruit underserved groups.^25^

The CTU survey highlighted ongoing gaps in the operationalisation of EDI strategies. Less than one-third of units had a designated EDI lead and only one in 10 reported a unit-wide EDI strategy. Most inclusivity efforts were focused on planning, preparation and trial setup, with less consistent application during trial conduct, analysis or dissemination. Targeted under-represented groups were largely limited to ethnic minorities, socio-economically disadvantaged participants and individuals with language barriers. Other under-represented groups, such as people with disabilities, refugees or age extremes, were less frequently considered. These findings show that, despite existing guidance frameworks, EDI practice remains inconsistent.

### Comparison with existing guidance

Unlike existing frameworks and toolkits, such as the NIHR INCLUDE guidelines, UK Health Research Authority (HRA) Inclusion and Diversity guidance and REP-EQUITY,^8 10 26^ the checklist is operational and actionable. It complements existing guidance and regulatory expectations by providing a practical framework that can be applied across different research settings and jurisdictions. The checklist is not intended to replace effective interventions or address resource constraints, but to help researchers systematically identify opportunities for inclusion and translate existing recommendations into actionable considerations. It is also a living document which will be updated with examples of good practice and case studies. Many of the existing guidelines were also developed using robust methodology, including scoping reviews, community engagement and consensus processes. However, these guidelines provide only high-level recommendations, while our checklist breaks these into concrete steps that researchers can implement, monitor and document. Its design mirrors the success of consensus-based reporting tools in other domains, such as CONSORT and PRISMA, which have demonstrably improved reporting quality.^17 19^ The checklist also incorporates flexibility to reflect trial-specific contexts, including optional items such as decentralised trial methods and partial participation options, enabling adaptation to diverse research designs.

### Evaluation of the inclusivity checklist

The inclusivity checklist is being evaluated in multiple studies in the National Cardiac Surgery Trials Initiative: DECIDE-TAD (NIHR206798); FitforHeartOp (NIHR207502); BCIS-4 (NIHR155123); DECIDE-CAD (NIHR304685). The evaluation will include surveys with users, qualitative interviews / focus groups with researchers to explore experiences and collecting recruitment metrics from individual trials

### Strengths and limitations of this study

The checklist was developed using robust methods, involving iterative refinement with formal consensus methods. The checklist includes clear statements, appendices, examples and case studies, making it immediately usable in trial planning and delivery. We had input from researchers, clinicians, funders, EDI and PPI experts and community members. This breadth ensured the checklist reflects multiple perspectives and practical realities. Under-served communities and PPI were at the core of the development process: half of the participants at the consensus workshop were patients or public members, including individuals from the community outreach activities. This enabled meaningful co-production and empowered participants to actively contribute to decision-making. As one public member commented: *“*I must say I was impressed by your ability to connect with the public on the ground, whilst being able to actively listen. It’s not often seldom-heard voices get to be heard, but they were”. The checklist is formally registered with the EQUATOR Network, supporting transparency and uptake.^27^ The checklist is currently being evaluated in several ongoing trials, allowing assessment of feasibility, acceptability and impact on recruitment of under-represented groups. The checklist is also being used to support the development of a set of comprehensive communication strategies in recruitment and retention. These are based on behavioural theories on communication and are being co-produced by the Leicester Clinical Trials Unit and the National Cardiac Surgery Clinical Trials Initiative, together with PPI members, healthcare professionals, clinical trialists and other stakeholders.

The checklist has several limitations. First, our scoping review was limited to MEDLINE and English-language publications and used a pragmatic saturation criterion; it is therefore possible that we may have missed some relevant studies. However, we anticipate that the identified strategies capture the majority of commonly employed approaches, given the finite nature of inclusion strategies in adult clinical research. The CTU survey had a 41% CTU response rate, and CTUs with a particular interest in EDI may have been more likely to participate, so findings may overestimate the extent to which EDI strategies are currently considered. Time and resource constraints prevented follow-up with non-responding units. Our community outreach drew on a small number of groups in and around Leicester and did not include all types of under-served populations, which limits generalisability. For example, some ethnic groups, traveller communities, people with sensory impairments, younger age groups, single parents, shift workers, Lesbian, Gay, Bisexual, Transgender, Queer/Questioning and Plus, etc were not represented. Consequently, the checklist may not fully address the unique barriers faced by these populations. However, the iterative development through the scoping review, survey and community engagement across different stakeholders would have mitigated this influence. Furthermore, we plan the checklist to be a living document, which will be updated with case-studies and evidence-based strategies in any under-represented group, as the evidence accumulates.

The checklist was initially developed with a focus on cardiovascular studies in secondary care. While many principles are generalisable, adaptation may be required for primary care, social care or paediatric research settings. Finally, although the checklist has face and content validity through multi-stakeholder consensus, we have not yet evaluated its reliability, feasibility in routine use or impact on recruitment and reporting of under-served groups. These are important priorities for future work.

### Implications for research and practice

This checklist provides an operational framework for embedding inclusivity throughout the research process. By encouraging systematic documentation, it can improve transparency, enable evaluation of strategies and facilitate replication across trials. Its use in ongoing studies will provide data on feasibility and impact, which will allow the checklist to evolve as a living document, incorporating case studies and examples of good practice over time.

The checklist also addresses a critical gap in current guidance: the lack of practical tools to translate high-level EDI principles into trial-specific actions. While existing frameworks outline ‘what’ should be considered, the checklist guides researchers on ‘how’ to implement inclusive practices, from budgeting for translation services to planning flexible participation options. It also highlights that funders need to commit sufficient and realistic funding to cover the cost of embedding inclusivity across the entire research pathway.

## Conclusion

The inclusivity checklist provides a practical, evidence-informed and consensus-based tool for improving equity in clinical research. It bridges the gap between principle and practice, operationalising inclusivity considerations from trial design to dissemination. Through ongoing evaluation and refinement, it has the potential to standardise inclusive practices, reduce disparities in research participation and enhance the generalisability of clinical study findings.

## Supplementary material

10.1136/bmjopen-2026-127636 online supplemental file 1

## Data Availability

All data relevant to the study are included in the article or uploaded as supplementary information.

## References

[1] Khan SU, Khan MZ, Raghu Subramanian C (2020). Participation of Women and Older Participants in Randomized Clinical Trials of Lipid-Lowering Therapies: A Systematic Review. JAMA Netw Open.

[2] Xiao H, Vaidya R, Liu F (2023). Sex, Racial, and Ethnic Representation in COVID-19 Clinical Trials. JAMA Intern Med.

[3] Unger JM, Cook E, Tai E (2016). The Role of Clinical Trial Participation in Cancer Research: Barriers, Evidence, and Strategies. Am Soc Clin Oncol Educ Book.

[4] Oh SS, Galanter J, Thakur N (2015). Diversity in Clinical and Biomedical Research: A Promise Yet to Be Fulfilled. PLoS Med.

[5] Filbey L, Zhu JW, D’Angelo F (2023). Improving representativeness in trials: a call to action from the Global Cardiovascular Clinical Trialists Forum. Eur Heart J.

[6] Clark LT, Watkins L, Piña IL (2019). Increasing Diversity in Clinical Trials: Overcoming Critical Barriers. Curr Probl Cardiol.

[7] Funding NIoHG (2001). NIH policy and guidelines on the inclusion of women and minorities as subjects in clinical research. https://grants.nih.gov/policy-and-compliance/policy-topics/inclusion/women-and-minorities.

[8] Research NIfHaC (2020). Improving inclusion of under-served groups in clinical research: guidance from the NIHR-INCLUDE project.

[9] Biggs K, Treweek S, Lane A STEP up: strategies for trialists to promote equal participation in clinical trials for under-served populations.

[10] Retzer A, Ciytak B, Khatsuria F (2023). A toolkit for capturing a representative and equitable sample in health research. Nat Med.

[11] U.S. Food and Drug Administration (2018). Pregnant women: scientific and ethical considerations for inclusion in clinical trials guidance for industry.

[12] U.S. Food and Drug Administration (2023). Pediatric drug development: regulatory considerations — complying with the pediatric research equity act and qualifying for pediatric exclusivity under the best pharmaceuticals for children act.

[13] U.S. Food and Drug Administration (2024). Diversity action plans to improve enrollment of participants from underrepresented populations in clinical studies.

[14] Collister D, Song C, Ruzycki SM (2024). Fostering diversity in clinical trials: need for evidence and implementation to improve representation. BMJ Med.

[15] Hales BM, Pronovost PJ (2006). The checklist--a tool for error management and performance improvement. J Crit Care.

[16] Winters BD, Gurses AP, Lehmann H (2009). Clinical review: checklists - translating evidence into practice. Crit Care.

[17] Page MJ, McKenzie JE, Bossuyt PM (2021). The PRISMA 2020 statement: an updated guideline for reporting systematic reviews. BMJ.

[18] Moher D, Hopewell S, Schulz KF (2010). CONSORT 2010 explanation and elaboration: updated guidelines for reporting parallel group randomised trials. BMJ.

[19] Moher D, Schulz KF, Simera I (2010). Guidance for developers of health research reporting guidelines. PLoS Med.

[20] Bodicoat DH, Routen AC, Willis A (2021). Promoting inclusion in clinical trials-a rapid review of the literature and recommendations for action. Trials.

[21] Pufulete M, Mahadevan K, Johnson TW (2022). Confounders and co-interventions identified in non-randomized studies of interventions. J Clin Epidemiol.

[22] Madurasinghe VW, Bower P, Eldridge S (2021). Can we achieve better recruitment by providing better information? Meta-analysis of “studies within a trial” (SWATs) of optimised participant information sheets. BMC Med.

[23] Dawson S, Banister K, Biggs K (2022). Trial Forge Guidance 3: randomised trials and how to recruit and retain individuals from ethnic minority groups-practical guidance to support better practice. Trials.

[24] Biggs K, Dix C, Shiely F (2024). Effective interventions to increase representation of under-served groups in randomised trials in UK and Ireland: a scoping literature review. *NIHR Open Res*.

[25] Parker A, Way R, Okanlawon AA (2024). WP1: identifying and prioritising trial recruitment and retention strategies.

[26] NHS Health Research Authority (2025). Guidance for developing and submitting an inclusion and diversity plan second draft. https://www.hra.nhs.uk/about-us/news-updates/guidance-developing-and-submitting-inclusion-and-diversity-plan-second-draft/.

[27] (2025). Inclusivity checklist for clinical research enhancing the quality and transparency of health research (equator network). https://www.equator-network.org/library/reporting-guidelines-under-development/reporting-guidelines-under-development-for-observational-studies/#Inclusivity.

