# Supplementary material for: Co-production of a consensus-based inclusivity checklist to promote inclusion and diversity in clinical research: a multi-method study
Source: BMJ Open. 2026 Oct 27;16(10):e127636. doi: 10.1136/bmjopen-2026-127636 (PMC13723870; doi:10.1136/bmjopen-2026-127636)
Supplement: Supplementary file 1 [file bmjopen-2026-127636.reviewer_comments.pdf]

PEER REVIEW HISTORY

BMJ Open publishes all reviews undertaken for accepted manuscripts. Reviewers are asked to complete a checklist review form and are provided with free text boxes to elaborate on their assessment. These free text comments are reproduced below.

This paper was submitted to another journal from BMJ but declined for publication following peer review. The authors addressed the reviewers' comments and submitted the revised paper to BMJ Open. The paper was subsequently accepted for publication at BMJ Open.

ARTICLE DETAILS

Title (Provisional)

The co-production of a consensus-based inclusivity checklist to promote inclusion and diversity in clinical research: a multi-method study

Authors

Cheng, Ann; Aujla, Hardeep; Grant, Robert; Mowete, Chibuzo; Alhashimi, Ali; Dearling, Jeremy; Murray, Sarah; Page, Susan; Mukuka, Esther; Murphy, Gavin; Pufulete, Maria; National Cardiac Surgery Clinical Trials Programme, Equality, Diversity and Inclusion Group

---

VERSION 1 - REVIEW

**Reviewer**	**1**
**Name**	**Ruzycki, Shannon**
**Affiliation**	**University of Calgary, Department of Medicine**
**Date**	**06-Mar-2026**
**COI**	**None**

The authors have identified and worked to address a massive challenge (underrepresentation of marginalized people in clinical research) with a pragmatic solution (checklists) using rigorous methods. The limitations of their methods are well described and are inherent to the limitations in current knowledge around this topic.

Despite these strengths, there was information missing from the objective and methods that would help readers understand how the checklist was created and how it can be used. Specific methodologic details are needed before the manuscript can be published.

The authors could be more explicit about how and when this checklist should be used in the "Objective" paragraph of the Introduction. For example, they mention that the checklist

should be used by funders - but how? To evaluate proposals? This comes up for me again later, when reading the checklist - statements like "Document the diversity of the research team" are unclear (Document this where? To what end?). Is this intended to be a reporting checklist in the final manuscript?

I am unfamiliar with the stopping rule that the authors used for determining that they had completed identification of articles for the scoping review (no new information after 10 papers) - is this standard? Is there supporting evidence for this method, or is this a pragmatic choice for living reviews?

The authors state that they "worked iteratively to categorise data into themes based on our conceptual framework" - does this framework mean the socioecologic levels or the research lifecycle? Or something else that I have missed? Looking at the supplementary tables it seems that the data was group by research cycle stage, which I would not classify as either a theme or a conceptual framework. Who is "we" who themed this data? Was it the steering committee or the scoping review team (4 reviewers)?

Draft checklist development: It sounds like the authors used a form of intervention mapping to match barriers to potential facilitators. Was a specific method followed? Who was involved in this process? Beyond the socioecologic framework introduced in the Methods (individual, societal, etc), was a formal implementation framework used to match barriers to solutions?

The authors state that they made "strategic" decisions about what to include and exclude from the checklist - but what were specific criteria used to determine whether a decision was strategic (e.g., acceptability, feasibility?)? What expertise or opinions were included when determining whether an item should be excluded or not?

For the one-day consensus workshop, was a specific consensus-building method used? How was consensus reached? How was dissent or disagreement managed? Was a majority-rules approach used, and if so, how were minority or marginalized perspectives amplified?

After looking at the survey questions, I wasn't sure how the survey results informed the checklist development. They seem more like an environmental scan of current practices rather than an instrument to develop new checklist items. In the supplementary table 6, none of the items were generated from the survey. The authors should be more explicit about the purpose of the survey considering the questions used and how that informed the overall project. Since most of the survey questions were developed using the scoping review, how was new information generated from the survey to inform the checklist? Overall, was the data from the survey helpful in developing the checklist and if so, how?

At first, I thought the checklist was to be used in reporting (similar to EQUATOR guidelines) but in reading the checklist, I am not sure - for example, where should study teams "Document the diversity of the research team"? Is this in the funding application? I am also worried about the power imbalance on a research team (between PI and trainees, for example) and how this may interact with need to disclose sensitive identities (e.g., LBGTQ+ status). Similarly, I wonder about the drawbacks of collecting comprehensive demographic data about participants - couldn't over-collection of certain demographics risk a loss of anonymity?

As presented, Table 2 is long and unsynthesized. The authors should present the most useful data from the surveys and perhaps use this table to demonstrate how the data was used in the final checklist rather than just listing responses.

The Discussion paragraph about the survey implies that a designated EDI lead is important for trial diversity but this is not an item on the checklist.

**Reviewer**	**2**
**Name**	**Reviewer 2**
**Affiliation**	
**Date**	**06-Mar-2026**
**COI**	

Comments not published pending reviewer’s consent

**Reviewer**	**3**
**Name**	**Reviewer 3**
**Affiliation**	
**Date**	**06-Mar-2026**
**COI**	

Comments not published pending reviewer’s consent

**Reviewer**	**4**
**Name**	**Reviewer 4**
**Affiliation**	
**Date**	**06-Mar-2026**

COI

Comments not published pending reviewer’s consent

VERSION 1 - AUTHOR RESPONSE

Reviewer: 1

The authors have identified and worked to address a massive challenge (underrepresentation of marginalized people in clinical research) with a pragmatic solution (checklists) using rigorous methods. The limitations of their methods are well described and are inherent to the limitations in current knowledge around this topic.

Despite these strengths, there was information missing from the objective and methods that would help readers understand how the checklist was created and how it can be used. Specific methodologic details are needed before the manuscript can be published.

2.1 The authors could be more explicit about how and when this checklist should be used in the "Objective" paragraph of the Introduction. For example, they mention that the checklist should be used by funders - but how? To evaluate proposals? This comes up for me again later, when reading the checklist - statements like "Document the diversity of the research team" are unclear (Document this where? To what end?). Is this intended to be a reporting checklist in the final manuscript?

We thank the reviewer for highlighting this ambiguity. We agree that the intended timing and practical application of the checklist should be clearer. We have therefore revised the Objective paragraph and the checklist instructions to specify that the checklist is intended primarily as a practical tool for research teams to consider and document inclusivity throughout the research lifecycle, from study planning and design through recruitment, delivery, data collection and dissemination. It can also be used at the reporting stage to document the inclusivity activities undertaken and identify areas where these were not possible.

The checklist is not intended to function solely as a reporting checklist. We envisage that researchers can use it prospectively when developing a study or protocol, and that funders, CTUs and other research stakeholders may use it to support review of proposals and study plans and to encourage consideration of inclusion at an early stage. We have clarified that its use should be adapted to the study context rather than treated as a mandatory set of requirements.

2.2 I am unfamiliar with the stopping rule that the authors used for determining that they had completed identification of articles for the scoping review (no new information after 10 papers) - is this standard? Is there supporting evidence for this method, or is this a pragmatic choice for living reviews?

See also response to BMJ Committee feedback (point 1.5). The use of 10 consecutive papers is not a universally standardised stopping rule; it is a pragmatic application of the broader concept of saturation, which is well established in qualitative research and has been applied in scoping/systematic reviews.[2] Our rationale was that the review sought to identify categories of barriers, facilitators and strategies to improve research inclusion rather than estimate their frequency. These are conceptually bounded and recurring constructs; the objective was therefore to identify the breadth of relevant concepts rather than exhaustively enumerate every publication describing them. Once 10 consecutive randomly selected studies yielded no new barriers or strategies, we considered that further extraction was unlikely to materially expand the conceptual framework. We have clarified that this was a pragmatic saturation criterion rather than a formally validated threshold and have added the supporting references.

2.3 The authors state that they "worked iteratively to categorise data into themes based on our conceptual framework" - does this framework mean the socioecologic levels or the research lifecycle? Or something else that I have missed? Looking at the supplementary tables it seems that the data was group by research cycle stage, which I would not classify as either a theme or a conceptual framework. Who is "we" who themed this data? Was it the steering committee or the scoping review team (4 reviewers)?

The conceptual framework used in this work is described in “Conceptual framework” under methods. We recognise that inclusivity can be framed both by socioecological levels and the research lifecycle. Supplemental table S6, which we believe the reviewer is referring to, highlights the conceptual framework where socioecological levels (e.g. participant mistrust, investigator bias, lack of organisational guidance, inadequate research infrastructure) and research procedures (e.g. research question, trial design, recruitment, dissemination) both present barriers. The data was thematised by the working group (AC, HA, RG, MP).

2.4 Draft checklist development: It sounds like the authors used a form of intervention mapping to match barriers to potential facilitators. Was a specific method followed? Who was involved in this process? Beyond the socioecologic framework introduced in the Methods (individual, societal, etc), was a formal implementation framework used to match barriers to solutions?

We did not use a formal implementation framework or intervention-mapping methodology to match barriers to solutions. Instead, we used the socioecological framework described in the Methods to organise the evidence by level (participant, researcher, organisational and societal) and iteratively mapped the identified barriers, facilitators and strategies to potential checklist items.

This process was undertaken by the Working Group, drawing on the scoping review, survey findings, community engagement and existing inclusion frameworks. The resulting checklist items were subsequently refined through iterative review by the Steering Group and final consensus workshop.

We did not consider a formal implementation framework necessary because the objective was not to develop or evaluate an implementation intervention, but to identify practical

opportunities for improving inclusion across the research lifecycle and translate these into a consensus-based checklist. We have clarified this distinction and the role of the Working Group in draft checklist development in the revised Methods.

2.5 The authors state that they made "strategic" decisions about what to include and exclude from the checklist - but what were specific criteria used to determine whether a decision was strategic (e.g., acceptability, feasibility?)? What expertise or opinions were included when determining whether an item should be excluded or not?

Decisions regarding inclusion, exclusion and refinement of checklist items were made iteratively by the Steering Group and during the final consensus workshop. These decisions were informed by the evidence identified in the scoping review, existing inclusion frameworks, survey findings and community engagement, alongside the expertise and perspectives of the multidisciplinary stakeholder group.

We did not apply prespecified criteria such as feasibility or acceptability thresholds to individual items. This was intentional, as the purpose of the process was to co-produce a consensus-based checklist and allow stakeholders to consider the relevance and practical applicability of potential items across different research contexts. Applying rigid criteria at the outset could have constrained this deliberative process. Items were therefore discussed and refined iteratively, with the final content determined through stakeholder consensus rather than by the Working Group alone. We have clarified this process in the Methods section and replaced “strategic decisions” with more explicit wording describing the iterative consensus process.

2.6 For the one-day consensus workshop, was a specific consensus-building method used? How was consensus reached? How was dissent or disagreement managed? Was a majority-rules approach used, and if so, how were minority or marginalized perspectives amplified?

The one-day workshop followed a consensus development conference approach. No formal voting or majority-rules process was used; consensus was reached through facilitated discussion and agreement. Participants worked in breakout groups to discuss different sections of the checklist, with each group co-facilitated by PPI members. This structure enabled detailed discussion of individual checklist items and provided an opportunity for patient, public and community perspectives to inform the final content. Groups then fed back to the wider workshop, where issues and proposed changes were discussed collectively.

2.7 After looking at the survey questions, I wasn't sure how the survey results informed the checklist development. They seem more like an environmental scan of current practices rather than an instrument to develop new checklist items. In the supplementary table 6, none of the items were generated from the survey. The authors should be more explicit about the purpose of the survey considering the questions used and how that informed the overall project. Since most of the survey questions were developed using the scoping review, how was new information generated from the survey to inform the checklist? Overall, was the data from the survey helpful in developing the checklist and if so, how?

Thank you for highlighting our mistake with supplementary table 6. An incorrect version without selecting survey as an evidence source was used in the manuscript; this has now been corrected in Supplemental Material V1.1.

We agree that the survey provided an important environmental scan of current practice, but its purpose was broader. In addition to asking about current approaches to inclusion, the survey explored perceived barriers and facilitators to inclusion and strategies considered useful for addressing these.

The survey findings were therefore used to identify and prioritise the barriers and facilitators that were most relevant to research teams in practice and to inform the strategies considered for inclusion in the checklist. While the survey did not generate checklist items independently (as shown in Table S6), its findings were triangulated with the scoping review, community engagement and existing frameworks during checklist development. This helped ensure that the checklist addressed barriers encountered in practice and that proposed strategies were considered relevant and practical by those working within the UK clinical research environment.

We have clarified the purpose and contribution of the survey in the Methods section and now explicitly describe it as informing the prioritisation and refinement of checklist content, rather than as a standalone source of checklist items.

2.8 At first, I thought the checklist was to be used in reporting (similar to EQUATOR guidelines) but in reading the checklist, I am not sure - for example, where should study teams "Document the diversity of the research team"? Is this in the funding application? I am also worried about the power imbalance on a research team (between PI and trainees, for example) and how this may interact with need to disclose sensitive identities (e.g., LBGTQ+ status). Similarly, I wonder about the drawbacks of collecting comprehensive demographic data about participants - couldn't over-collection of certain demographics risk a loss of anonymity?

We consider the collection of relevant demographic information important because without understanding who participates, it is difficult to assess whether the populations intended to benefit from research are represented. However, this should be balanced against proportionality, privacy and participant choice. How this is operationalised should be determined by the research team according to the study context, available resources and the sensitivity of the information involved. We have clarified this in the checklist guidance.

Research participants are frequently asked to complete demographic questions and the same should apply to the research team. However, we would suggest that any demographic collection needs to include ‘prefer not to answer’ as an option to maintain anonymity as necessary.

2.9 As presented, Table 2 is long and unsynthesized. The authors should present the most useful data from the surveys and perhaps use this table to demonstrate how the data was used in the final checklist rather than just listing responses.

We agree that Table 2 is long and have moved the responses to Supplementary Material Table S4 instead. The current text within the manuscript provides a succinct description of the key results.

2.10 The Discussion paragraph about the survey implies that a designated EDI lead is important for trial diversity but this is not an item on the checklist.

The designated EDI lead is suggested by checklist item #7 “designate individual(s) responsible for delivering the Research Inclusion Plan”.

VERSION 2 - REVIEW

**Reviewer**	**1**
**Name**	**Ruzycki, Shannon**
**Affiliation**	**University of Calgary, Department of Medicine**
**Date**	**21-Sep-2026**
**COI**	

On my first review, I wondered about how the checklist would be used. The study team has clarified that it is for internal use for the research team to design and monitor their studies in their response to reviewers, though this is still not as explicit in the manuscript. I suggest explicitly stating that the checklist should be used within the study team to design and guide a study, with possible future applications for funders and other groups.

Though the study team responded to my question about who matched facilitators to barriers in the response to reviewers, I didn't see any update in the manuscript to describe the process beyond an example. The authors should clarify who performed this matching and how it was decided (e.g., using literature? expertise? best guess?)

I appreciated the information about the consensus development conference approach. Were there any minority opinions that couldn't be accommodated?

VERSION 2 - AUTHOR RESPONSE

Reviewer: 1

On my first review, I wondered about how the checklist would be used. The study team has clarified that it is for internal use for the research team to design and monitor their studies in their response to reviewers, though this is still not as explicit in the manuscript. I suggest explicitly stating that the checklist should be used within the study team to design and guide a study, with possible future applications for funders and other groups.

We had amended the objective to be more explicit as per reviewer 1’s previous suggestion. In addition, we have now also revised the manuscript at first paragraph under discussion to further highlight when the checklist should be used.

Though the study team responded to my question about who matched facilitators to barriers in the response to reviewers, I didn't see any update in the manuscript to describe the process beyond an example. The authors should clarify who performed this matching and how it was decided (e.g., using literature? expertise? best guess?)

We have amended the manuscript at Methods, Draft checklist development to clarify the process as suggested.

I appreciated the information about the consensus development conference approach. Were there any minority opinions that couldn't be accommodated?

The consensus workshop did not identify any minority or dissenting opinion regarding iterative refinements made to the checklist. We have added a sentence in the manuscript under Results, Checklist development to provide additional clarity.
