# Supplementary material for: Co-production of a consensus-based inclusivity checklist to promote inclusion and diversity in clinical research: a multi-method study
Source: BMJ Open. 2026 Oct 27;16(10):e127636. doi: 10.1136/bmjopen-2026-127636 (PMC13723870; doi:10.1136/bmjopen-2026-127636)
Supplement: Supplementary file 2 [file bmjopen-16-10-s001.docx]

# SUPPLEMENTAL MATERIAL

**Table S1**. Scoping review search strategy

**Table S2**. Scoping review data extraction form

**Table S3**. CTU questionnaire administered

**Table S4**. CTU questionnaire responses

**Table S5.** Description of communities involved in outreach

**Table S6.** Summary of barriers and facilitators identified, including evidence sources, which informed the first checklist draft

**Table S7.** Prototype Inclusivity Checklist (40-item) used at the Consensus Workshop

**Table S8.** Consensus workshop breakout group sessions

**Table S9.** Equality, Diversity and Inclusion Group of the National Cardiac Surgery Clinical Trials Initiative (Group Authorship)

**Table S10**. Inclusivity Checklist with full guidance

## Table S1. Scoping review search strategy

(underserved OR under-served OR underrepresented OR under-represented OR seldom-heard OR “seldom heard” OR “hard to reach” OR “hard-to-reach” OR minority OR minorities OR marginalised OR marginalized OR discriminated OR disability OR “mental incapacity” OR ethnicity OR gender OR migrant* OR “asylum seeker*” OR traveller* OR deprived OR race OR age OR “socio-economic status” OR “care home*” OR prison OR homeless)

AND

(challenge* OR barrier* OR facilitator* OR approach* OR strategy OR strategies)

AND

(divers* OR inclusi* OR equal*)

AND

(“clinical trial*” OR “controlled trial*”)

Blue colour denotes changes to the original search strategy published by Bodicoat et al. following discussion with the author herself.

## Table S2. Scoping review data extraction form

| First author | Journal | Year | Country | Study design | Study description | N | Underserved group(s) | Conditions | Barriers | Key strategies/enablers | Study results |
| --- | --- | --- | --- | --- | --- | --- | --- | --- | --- | --- | --- |
|  |  |  |  |  |  |  |  |  |  |  |  |

Strategies and barriers were extracted as individual items. For example,

*‘Strategies that attempt to overcome [logistical barriers] include flexible timings and locations of study visits, including home-based assessments; providing childcare; transport; and reducing costs associated with trial participation’ – Bodicoat et al 2021 on tackling logistical barriers*

were extracted as:

- Flexible timing
- Flexible study locations, including home visits
- Provision of childcare
- Provision of transport
- Cost reduction (unspecified)

## Table S3. CTU Questionnaire administered

Equality Diversity and Inclusion (EDI) in Clinical Trials Survey

Thank you for your interest in this survey. It will take you approximately 15 minutes to complete.

What this survey is about?

We want to develop and test an Equality, Diversity, and Inclusion (EDI) toolkit for cardiovascular trials. To help us with this process, we want to identify current EDI practice with regards to trial design, conduct, and reporting across all therapeutic areas (not just cardiovascular trials). We are therefore asking all UK clinical trials units (CTUs) to complete this survey. We also want to collect some demographic information about your CTU.

Who is conducting this survey?

This survey is part of a wider project being undertaken by a collaborative group of academic and patient researchers with representation from the University of Leicester and the University of Bristol.

Who should take part?

It is important to gather a broad range of opinions. We would therefore like to hear from any relevant CTU staff, including EDI leads, trial managers, methodologists, statisticians, directors, etc. Please forward this survey to anyone in your CTU who is currently designing, conducting or reporting a clinical trial of any type, in any disease area.

What will happen next?

The results of this survey will be used to inform further research. If you would like to participate in further research, please leave your email address at the end of this survey and we will contact you with further information on how you can help. This is optional and you can complete the current survey without leaving your email address.

This survey is provided via JISC Online Surveys at the University of Leicester. Your responses will be held for 5 years. All data will be stored on the University of Leicester and University of Bristol secure computing networks. Only pooled survey results will be reported. No CTU or individual will be identified in any report from this survey. Each participating CTU will receive a copy of the results.

If you have any questions, please contact Hardeep Aujla ([ha200@leicester.ac.uk](mailto:ha200@leicester.ac.uk)) or Maria Pufulete ([maria.pufulete@bristol.ac.uk](mailto:maria.pufulete@bristol.ac.uk)).

If you have read the prior, please indicate your consent to participate in the survey:

☐ I understand the project and am happy to consent

☐ I do not consent [automatically taken to the thank you page at end of the survey]

**********************************************************************************

About your CTU

Name of your CTU:

☐ Barts and the London Pragmatic CTU

☐ Barts Clinical Trials Unit

☐ Birmingham Clinical Trials Unit

☐ Brighton and Sussex Clinical Trials Unit

☐ Bristol Trials Centre

☐ CaCTUS (Cancer Clinical Trials Unit Scotland)

☐ Cambridge Clinical Trials Unit (CCTU)

☐ Cambridge Epidemiology & Trials Unit

☐ Cancer Research UK Clinical Trials Unit (CRCTU)

☐ Centre for Healthcare Randomised Trials (CHaRT)

☐ Centre for Trials Research (Cardiff)

☐ Comprehensive CTU @ UCL

☐ CRUK & UCL Cancer Trials Centre

☐ Derby Clinical Trials Support Unit (DCTSU)

☐ Diabetes Trials Unit, University of Oxford

☐ Edinburgh Clinical Trials Unit

☐ Exeter Clinical Trials Unit

☐ Glasgow Clinical Trials Unit

☐ Hull Health Trials Unit

☐ Imperial Clinical Trials Unit

☐ Intensive Care National Audit & Research Centre (ICNARC) CTU

☐ Keele Clinical Trials Unit

☐ King's Clinical Trials Unit at King's Health Partners

☐ Lancashire Clinical Trials Unit

☐ Leeds Clinical Trials Research Unit

☐ Leicester Clinical Trials Unit

☐ Liverpool Trials Collaborative

☐ London School of Hygiene & Tropical Medicine

☐ Medical Research Council Clinical Trials Unit at UCL

☐ Newcastle Clinical Trials Unit (NCTU)

☐ NHS Blood and Transplant Clinical Trials Unit

☐ North Wales Organisation for Randomised Trials in Health (NWORTH)

☐ Northern Ireland Clinical Trials Unit

☐ Norwich Clinical Trials Unit

☐ Nottingham Clinical Trials Unit

☐ NPEU Clinical Trials Unit

☐ Oxford Clinical Trial Service Unit & Epidemiological Studies Unit (CTSU)

☐ Oxford Clinical Trials Research Unit (OCTRU)

☐ Oxford Primary Care and Vaccines Collaborative Clinical Trials Unit

☐ Papworth Trials Unit Collaboration

☐ Peninsula Clinical Trials Unit

☐ PRIMENT Clinical Trials Unit at UCL

☐ Royal Marsden Clinical Trials Unit (RM-CTU)

☐ Sheffield Clinical Trials Research Unit

☐ Southampton Clinical Trials Unit

☐ Surrey Clinical Trials Unit

☐ Swansea Trials Unit

☐ Tayside Clinical Trials Unit

☐ The Institute of Cancer Research Clinical Trials & Statistics Unit (ICR- CTSU)

☐ Warwick Clinical Trials Unit

☐ York Trials Unit

☐ Other (please specify): ____________________________________________________

Your job title:

☐ Director or senior management

☐ Research or programme manager

☐ Trial management (trial manager or coordinator)

☐ Statistician

☐ Quality assurance

☐ IT or programmer

☐ Data manager

☐ Would rather not say

☐ Other (please specify): _________________________________________________

Approximately how many trials are currently active in your CTU? (i.e. awarded funding, in set up, recruitment, close down)

☐ ≤ 5

☐ 6-15

☐ 16-25

☐ 26-30

☐ ≥ 31

☐ Don’t know

☐ Other (please specify): ____________________________________________________

Which settings are your trial/s recruiting from? (select all that apply)

☐ Primary care

☐ Secondary care

☐ Tertiary care

☐ Social care

☐ Healthy volunteers

☐ Don’t know

☐ Other (please specify): ____________________________________________________

Approximately how many staff are currently employed in your CTU?

☐ ≤15

☐ 16-30

☐ 31-45

☐ 46-60

☐ ≥ 61

☐ Don’t know

☐ Other (please specify): ____________________________________________________

Do you think the staff working in your CTU represent a diverse range of backgrounds (e.g. with regards to age, gender, ethnicity, cultural background, etc.)

☐ Staff in my CTU are very representative

☐ Staff in my CTU are somewhat representative

☐ Staff in my CTU are not representative

☐ Don’t know

Comments: ________________________________________________________________

EDI strategies in trials

Is there a designated EDI representative / lead in your CTU?

☐ Yes

☐ No

☐ Don’t know

Comments: ________________________________________________________________

Is there a unit-wide EDI strategy in place to increase recruitment of people from under-represented groups in trials?

☐ Yes, we have a unit-wide strategy

☐ Yes, we are currently developing a unit-wide EDI strategy

☐ No, we currently don’t have a unit-wide EDI strategy

☐ Don’t know

Comments: ________________________________________________________________

Does you CTU apply the unit-wide EDI strategy to:

☐ Not applicable (we don’t have a unit-wide EDI strategy)

☐ All open trials

☐ Recently funded trials / trials in set-up

☐ If the funder / trial CI requests this

☐ Don’t know

☐ Other (please specify): ____________________________________________________

If you don’t have a unit-wide EDI strategy, do you consider EDI strategies in individual trials?

☐ Not applicable (we have a unit-wide EDI strategy)

☐ Yes, in all open trials

☐ Only in recently funded trials / trials in set-up

☐ Only if the funder / trial CI requests this

☐ We currently don’t consider EDI strategies in any of our trials

☐ Don’t know

☐ Other (please specify): ____________________________________________________

Is the EDI strategy (unit-wide strategy or any strategy applied at the individual trial level) informed by any existing guidance? (select all that apply)

☐ Not applicable (we don’t consider EDI strategies in any of our trials)

☐ NIHR-INCLUDE Guidelines

☐ NIHR Research Inclusion Strategy

☐ NIHR Race Equality Framework

☐ The MESSAGE Project

☐ INCLUDE Ethnicity Framework

☐ HRA and MHRA Inclusion and Diversity Plan

☐ NHS England Good Practice Guide for Researchers

☐ Internally-defined guidance

☐ Don’t know

☐ Other (please specify): ____________________________________________________

At which stage of the trial process do you apply the EDI strategy (unit-wide strategy or any strategy applied at the individual trial level)? (select all that apply)

☐ Not applicable (we don’t consider EDI strategies in any of our trials)

☐ Planning (e.g. proposal writing, grant application)

☐ Preparation (e.g. protocol/information sheet/consent form drafting)

☐ Setup (e.g. participating site training)

☐ Live (e.g. investigator/steering committee meetings)

☐ Analysis (e.g. analysing trial results by under-represented groups)

☐ Dissemination (e.g. trial reports, PPI summaries, publications)

☐ Don’t know

☐ Other (please specify): ____________________________________________________

If you apply an EDI strategy (unit-wide strategy or any strategy applied at the individual trial level)), which under-represented groups have you considered? (select all that apply)

☐ Not applicable (we don’t consider EDI strategies in any of our trials)

☐ Age extremes (under 18 and over 75) (depending on the context of the trial)

☐ Female/male sex (depending on the context of the trial)

☐ Women of childbearing age (depending on the context of the trial)

☐ Ethnic diversity

☐ LGBTQ+

☐ Socio-economically disadvantaged/unemployed

☐ People in alternative residential circumstances (e.g. residential care homes, prisons, the homeless and those of no fixed abode)

☐ Refugees/asylum seekers

☐ Digital exclusion/disadvantage

☐ People with mental health issues

☐ People with cognitive impairment

☐ People with physical disabilities

☐ People with language barriers

☐ Don’t know

☐ Other (please specify): ____________________________________________________

What are the main barriers (select up to 5) that you have experienced when recruiting more people from under-represented groups?

☐ The way the trial is designed (e.g. strict eligibility criteria, multiple trial-related visits)

☐ Participant level barriers (e.g. mistrust, fear)

☐ Unconscious bias from site teams who recruit participants

☐ Communication (e.g. language barriers, lack of interpreters and translated material, literacy)

☐ Accessibility for potential participants (e.g. transport, distance, caring responsibilities)

☐ Lack of guidance/training/skills in how to approach people from under-represented groups

☐ Lack of confidence about how to approach people from under-represented groups

☐ Recruiting site team do not have sufficient time to apply EDI strategies

☐ CTU team do not have sufficient time to apply EDI strategies

☐ Insufficient funding to implement EDI strategies adequately

☐ EDI is not a priority for CI / trial team

☐ EDI is not specified in the trial design

☐ Trial needs to recruit quickly therefore easiest potential participants are approached

☐ Other (please specify): ____________________________________________________

Thinking about the current trial(s) you are working on, which of the following EDI strategies have you used to increase recruitment of people from under-represented groups (select all that apply)

☐ Not applicable (we don’t consider EDI strategies in any of our trials)

☐ Translation of participant facing documents

☐ Interpreting services

☐ Travel costs (for participants, families or carers)

☐ Other expenses (to cover loss of earnings or childcare)

☐ Community champions / community engagement officers

☐ Incentive payments for participating in trial

☐ Cultural competency training for research staff

☐ Cultural competency training for trial unit team

☐ Recruitment videos, or information in easy to read or visual formats such as infographics

☐ Providing mobile phones, modems, etc. to prevent digital exclusion

☐ Setting recruitment targets

☐ Offering options for participation (e.g. online versus in person)

☐ Monitoring participant:prevalence ratio

☐ None of the above

☐ Don’t know

☐ Other (please specify): ____________________________________________________

In your experience, which EDI strategies (select up to 5) have been the most effective at increasing participation of under-represented groups?

☐ Not applicable (we don’t consider EDI strategies in any of our trials)

☐ Translation of participant facing documents

☐ Interpreting services

☐ Travel costs (for participants, families or carers)

☐ Other expenses (to cover loss of earnings or childcare)

☐ Community champions / community engagement officers

☐ Incentive payments for participating in trial

☐ Cultural competency training for research staff

☐ Cultural competency training for trial unit team

☐ Recruitment videos, or information in easy to read or visual formats such as infographics

☐ Providing mobile phones, modems, etc. to prevent digital exclusion

☐ Setting recruitment targets

☐ Offering options for participation (e.g. online versus in person)

☐ Monitoring participant:prevalence ratio

☐ None of the above

☐ Don’t know

☐ Other (please specify): ____________________________________________________

Please provide examples of what has worked particularly well, in which context (which under-represented group was included) and why: _________________________________________________________________________

Which of the following do you / people in your CTU cost for in your current grant applications (select all that apply)

☐ Not applicable (we don’t cost for EDI in any of our trials)

☐ Translated consent materials (e.g. consent form, PIL, information video)

☐ Translated questionnaires (paper and electronic)

☐ Interpreter services

☐ Extra time for staff to recruit and follow-up participants

☐ Engagement activities (e.g. costs to host an event with community groups)

☐ Translation of outputs (e.g. participant results leaflets or videos)

☐ Dissemination events

☐ Don’t know

☐ Other (please specify): ____________________________________________________

Thinking about your current trial(s), do you think the EDI budget adequately covers the activities required to recruit people from under-represented groups?

☐ Not applicable (we don’t cost for EDI in any of our trials)

☐ Definitely

☐ Very probably

☐ Possibly

☐ Probably not

☐ Definitely not

☐ Don’t know

Comments: ________________________________________________________________

Is anyone conducting research related to EDI (e.g. SWATs) in your CTU?

☐ Yes

☐ No

☐ Don’t know

Diversity in Patient and Public Involvement (PPI) groups

Thinking about the PPI group(s) for your current trial(s), how representative are its members (e.g. with regards to the demographic characteristics of the trial population)

☐ The PPI group(s) is very representative

☐ The PPI group(s) is somewhat representative

☐ The PPI group(s) is not representative

☐ I don’t have a PPI group

☐ Don’t know

Comments: ________________________________________________________________

If you would like to be contacted about participating further in our research, please leave your name and email address below:

Name: __________________________________________________________

E-mail: __________________________________________________________

Thank you for completing this survey.

## Table S4. CTU Questionnaire responses

Please note place names have been anonymised

| **Survey question** | **N (%) endorsing different response categories**  **N=37** | |
| --- | --- | --- |
| *Your job title* | | |
| Statistician | 3 (8.1%) | |
| Quality Assurance | 1 (2.7%) | |
| Director or Senior Management | 7 (18.9%) | |
| Research or Programme Manager | 1 (2.7%) | |
| Trial Management (Trial Manager or Coordinator) | 17 (45.9%) | |
| Data Manager | 1 (2.7%) | |
| IT or Programmer | 1 (2.7%) | |
| Other  Clinician and Chief Investigator  Trial Monitor  Researcher in Inclusivity / Diversity & Inclusion Officer | 1 (2.7%)  2 (5.4%)  2 (5.4%) | |
| Would rather not say | 1 (2.7%) | |
| *Approximately how many trials are currently active in your CTU? (i.e. awarded funding, in set up, recruitment, close down)* | | |
| ≤ 5 | 2 (5.4%) | |
| 6 – 15 | 6 (16.2%) | |
| 16 – 25 | 2 (5.4%) | |
| 26 – 30 | 6 (16.2%) | |
| ≥ 31 | 16 (43.2%) | |
| Don’t know | 5 (13.5%) | |
| Other | 0 (0.0%) | |
| *Which settings are your trial(s) recruiting from?* | | |
| Primary care | 29 (78.0%) | |
| Secondary care | 31 (84.0%) | |
| Tertiary care | 13 (35.0%) | |
| Social care | 10 (27.0%) | |
| Healthy volunteers | 9 (24.0%) | |
| Don’t know | 4 (11.0%) | |
| Other  Community recruitment | 1 (3.0%) | |
| *Approximately how many staff are currently employed in your CTU?* | | |
| ≤ 15 | 1 (3.0%) | |
| 16 – 30 | 6 (16.0%) | |
| 31 – 45 | 2 (5.0%) | |
| 46 – 60 | 3 (8.0%) | |
| ≥ 61 | 22 (59.0%) | |
| Don’t know | 3 (8.0%) | |
| Other | 0 (0.0%) | |
| *Do you think the staff working in your CTU represent a diverse range of backgrounds (e.g. with regards to age, gender, ethnicity, cultural background, etc.)* | | |
| Staff in my CTU are very representative | 10 (27.0%) | |
| Staff in my CTU are somewhat representative | 23 (62.0%) | |
| Staff in my CTU are not representative | 2 (5.0%) | |
| Don’t know | 2 (5.0%) | |
| Comments:  “Not sure what 'representative' means here...? Of UK population / [name of place] population...”  “More females than males but not a huge discrepancy.”  “What do you mean by 'representative'? Representative of what?”  “We have representation from a few ethnic minority groups, gender minorities, someone with a disability, neurodiverse staff and a good gender balance (though mainly female) across a range of ages.”  “Lack of ethnicity or cultural background.”  “Gender imbalance.”  “Not sure how to define "representative" as these responses don't match the question asked. Does it mean exact proportions of ethnicity, gender according to the local population/UK population? As we have more women than men for example, so not "representative". But if the responses were "Staff in my CTU represent a diverse range of backgrounds", "Staff represent a somewhat diverse range of backgrounds"... then I could answer the question.”  “Very difficult to tell for sure as I don't have access to confidential personal characteristics of our staff.” | | |
| *Is there a designated EDI representative / lead in your CTU?* | | |
| Yes | 11 (30.0%) | |
| No | 13 (35.0%) | |
| Don’t know | 13 (35.0%) | |
| Comments:  “We are aware of the issue in particular that trials are representative of the areas they are being conducted in.”  “We have a dedicated Patient Public Involvement & Engagement Group but not aware of a dedicated EDI lead.”  “EDI linkage is currently at the institutional level through a designated EDI unit given the current size of the CTU.”  “We have an EDI working group and a number of members of staff with an interest in EDI, but no named EDI lead currently.”  “We have colleagues with particular expertise/experience in EDI, but no one person is designated as EDI lead.”  “There isn't one single individual as EDI lead, but there is a specific group on Inclusivity in Trials/Studies and an EDI committee (from a staff/culture perspective) both of which have leads.”  “The previous lead has just left post but will be replaced.” | | |
| *Is there a unit-wide EDI strategy in place to increase recruitment of people from under-represented groups in trials?* | | |
| Yes, we have a unit-wide strategy | 4 (11.0%) | |
| Yes, we are currently developing a unit-wide strategy | 8 (22.0%) | |
| No, we currently don’t have a unit-wide strategy | 13 (35.0%) | |
| Don’t know | 12 (32.0%) | |
| Comments:  “We have a dedicated Patient Public Involvement & Engagement Group but not aware of a dedicated EDI lead and strategy to implement this.”  “There is an institutional wide EDI unit that works with the people’s team that advances the EDI agenda when looking to recruit new personnel to the unit (recruitment is a centralised process).”  “We have discussed a unit wide strategy and have incorporated things into existing SOPs but not written an EDI strategy or policy.”  “After discussion, current approach is that EDI should be embedded across all strategies, processes, etc rather than separating out.”  “We don't have a formal separate EDI strategy but have integrated EDI guidance throughout all of our processes.” | | |
| *Does your CTU apply the unit-wide EDI strategy to:* | | |
| Not applicable (we don’t have a unit-wide EDI strategy) | 13 (35.0%) | |
| All open trials | 3 (8.0%) | |
| Recently funded trials / trials in set-up | 4 (11.0%) | |
| If the funder / trial CI requests this | 1 (3.0%) | |
| Don’t know | 13 (35.0%) | |
| Other | 3 (8.0%) | |
| Comments, if Other:  “As the policy is in set-up, we are applying this where we can at the moment whilst it is developed.”  “We apply it to all trials.”  “Encouraging consideration across all of our trials whatever stage they are at, implementing updated SOPs and processes at relevant stage (eg publication policy for trials undergoing analysis, implementation of detailed demographic data collection in all new trials and those relatively early in recruitment where possible).” | | |
| *If you don’t have a unit-wide EDI strategy, do you consider EDI strategies in individual trials?* | | |
| Not applicable (we don’t have a unit-wide EDI strategy) | 5 (14.0%) | |
| Yes, in all open trials | 8 (22.0%) | |
| Only in recently funded trials / trials in set-up | 11 (30.0%) | |
| Only if the funder / trial CI requests this | 3 (8.0%) | |
| We currently don’t consider EDI strategies in any of our trials | 1 (3.0%) | |
| Don’t know | 7 (18.0%) | |
| Other | 2 (5.0%) | |
| Comments, if Other:  “Consider improving EDI in active trials, and ensuring EDI in all facets of recently funded studies.”  “We are encouraging all trials to consider via SOP/process updates based on national recommendations, but there is little evidence on validated interventions to support/improve diversity to implement across all trials - we are focusing on data collection/assessment as a (no-cost) starting point, with EDI related SWATs embedded/planned in various newer trials. Keen to assess impact of EDI-related interventions so we can implement what works in any groups we confirm are underrepresented in any given trial.” | | |
| *Is the EDI strategy (unit-wide strategy or any strategy applied at the individual trial level) informed by any existing guidance? (select all that apply)* | | |
| Not applicable (we don’t have a unit-wide EDI strategy) | 2 (5.0%) | |
| NIHR-INCLUDE Guidelines | 16 (43.0%) | |
| NIHR Research Inclusion Strategy | 7 (19.0%) | |
| NIHR Race Equality Framework | 4 (11.0%) | |
| The MESSAGE Project | 3 (8.0%) | |
| INCLUDE Ethnicity Framework | 13 (35.0%) | |
| HRA and MHRA Inclusion and Diversity Plan | 8 (22.0%) | |
| NHS England Good Practice Guide for Researchers | 5 (14.0%) | |
| Internally-defined guidance | 7 (19.0%) | |
| Don’t know | 11 (30.0%) | |
| Other | 4 (11.0%) | |
| Comments, if Other:  “HRA-Patient Centred Clinical Research Outputs (Hallmarks & Recommendations) given responder was a member of the steering group.”  “Equality Impact Assessment toolkit.”  “[place name]”  “Other INCLUDE frameworks e.g socio-economic, impaired capacity to consent.” | | |
| *At which stage of the trial process do you apply the EDI strategy (unit-wide strategy or any strategy applied at the individual trial level)? (select all that apply)* | | |
| Not applicable (we don’t have a unit-wide EDI strategy) | 1 (3.0%) | |
| Planning (e.g. proposal writing, grant application) | 27 (73.0%) | |
| Preparation (e.g. protocol/information sheet/consent form drafting) | 28 (76.0%) | |
| Setup (e.g. participating site training) | 25 (68.0%) | |
| Live (e.g. investigator/steering committee meetings) | 20 (54.0%) | |
| Analysis (e.g. analysing trial results by under-represented groups) | 15 (41.0%) | |
| Dissemination (e.g. trial reports, PPI summaries, publications) | 17 (46.0%) | |
| Don’t know | 5 (14.0%) | |
| Other | 0 (0.0%) | |
| *If you apply an EDI strategy (unit-wide strategy or any strategy applied at the individual trial level), which under-represented groups have you considered? (select all that apply)* | | |
| Not applicable (we don’t have a unit-wide EDI strategy) | 1 (3.0%) | |
| Age extremes (under 18 and over 75) (depending on the context of the trial) | 10 (27.0%) | |
| Female/male sex (depending on the context of the trial) | 15 (41.0%) | |
| Women of childbearing age (depending on the context of the trial) | 6 (16.0%) | |
| Ethnic diversity | 24 (65.0%) | |
| LGBTQ+ | 7 (19.0%) | |
| Socio-economically disadvantaged / unemployed | 21 (57.0%) | |
| People in alternative residential circumstances (e.g. residential care homes, prison, the homeless and those of no fixed abode) | 6 (16.0%) | |
| Refugees / asylum seekers | 2 (5.0%) | |
| Digital exclusion / disadvantage | 18 (49.0%) | |
| People with mental health issues | 8 (22.0%) | |
| People with cognitive impairment | 9 (24.0%) | |
| People with physical disabilities | 7 (19.0%) | |
| People with language barriers | 19 (51.0%) | |
| Don’t know | 9 (24.0%) | |
| Other | 2 (5.0%) | |
| Comments, if Other:  “It will always depend on the context of the trial and aims to consider inclusivity in its widest sense but with consideration of particular groups, rather than a specific list which doesn't take into account intersectionality etc.”  “We ask researchers to build around the populations their trial needs to serve, so identifying which populations are underserved, although we have explicit guidance about certain groups too.” | | |
| *What are the main barriers (select up to 5) that you have experienced when recruiting more people from under-represented groups?* | | |
| The way the trial is designed (e.g. strict eligibility criteria, multiple trial-related visits) | 14 (38.0%) | |
| Participant level barriers (e.g. mistrust, fear) | 17 (46.0%) | |
| Unconscious bias from site teams who recruit participants | 7 (19.0%) | |
| Communication (e.g. language barriers, lack of interpreters and translated material, literacy) | 24 (65.0%) | |
| Accessibility for potential participants (e.g. transport, distance, caring responsibilities) | 15 (41.0%) | |
| Lack of guidance / training / skills in how to approach people from under-represented groups | 7 (19.0%) | |
| Lack of confidence about how to approach people from under-represented groups | 3 (8.0%) | |
| Recruiting site team do not have sufficient time to apply EDI strategies | 9 (24.0%) | |
| CTU team do not have sufficient time to apply EDI strategies | 5 (14.0%) | |
| Insufficient funding to implement EDI strategies adequately | 16 (43.0%) | |
| EDI is not a priority for CI / trial team | 0 (0.0%) | |
| EDI is not specified in the trial design | 6 (16.0%) | |
| Trial needs to recruit quickly therefore easiest potential participants are approached | 9 (24.0%) | |
| Other | 4 (11.0%) | |
| Comments, if Other:  “I do not have the data to support a top 5 for this. It is not always clear why, even when strategies have been put in place, the reasons why a more diverse population have not been recruited and would benefit more time to explore on a trial by trial basis.”  “Given the broad portfolio, I am not sure it is possible to select main barriers as they are very contextual - e.g a decentralised trial with older people being delivered remotely will encounter very different barriers to one delivered in person in prisons.”  “Location of sites.”  “Lack of robust data to identify which groups are not being recruited so we can target and address any barriers - insufficient resource to implement multiple non-validated interventions to blanket cover speculated lack of participation from any given group (we are implementing more detailed demographic data collection to address this).” | | |
|  | *30. Thinking about the current trial(s) you are working on, which of the following EDI strategies have you used to increase recruitment of people from under-represented groups* | *32. In your experience, which EDI strategies have been the most effective at increasing participation of under-represented groups?* |
| Not applicable (we don’t consider EDI strategies in any of our trials) | 2 (5.0%) | 1 (3.0%) |
| Translation of participant facing documents | 25 (68.0%) | 12 (32.0%) |
| Interpreting services | 18 (49.0%) | 8 (22.0%) |
| Travel costs (for participants, families or carers) | 24 (65.0%) | 9 (24.0%) |
| Other expenses (to cover loss of earnings or childcare) | 5 (14.0%) | 1 (3.0%) |
| Community champions / community engagement officers | 7 (19.0%) | 2 (5.0%) |
| Incentive payments for participating in trial | 20 (54.0%) | 7 (19.0%) |
| Cultural competency training for research staff | 3 (8.0%) | 0 (0.0%) |
| Cultural competency training for trial unit team | 5 (14.0%) | 0 (0.0%) |
| Recruitment videos, or information in easy to read or visual formats such as infographics | 24 (65.0%) | 11 (30.0%) |
| Providing mobile phones, modems, etc. to prevent digital exclusion | 10 (27.0%) | 1 (3.0%) |
| Setting recruitment targets | 4 (11.0%) | 0 (0.0%) |
| Offering options for participation (e.g. online versus in-person | 14 (38.0%) | 6 (16.0%) |
| Monitoring participant:prevalence ratio | 6 (16.0%) | 0 (0.0%) |
| None of the above | 0 (0.0%) | 0 (0.0%) |
| Don’t know | 1 (3.0%) | 14 (38.0%) |
| Other | 3 (8.0%) | 3 (8.0%) |
| Comments, if Other:  “Segregating PPIE groups by ethnicity.”  “Identifying and opening sites in more diverse areas; actively requesting applications from under represented groups for our PPI group.”  “Planning to implement some of these but lack of guidance and dedicated funding makes it difficult to change practice quickly (eg responsibility for interpretation has historically been with the NHS recruiting sites and we know translation of patient docs alone won't address language barrier issues).”  “Recruiting outside of usual care pathways.”  “Choosing the right sites/locations method to recruit. “  “Given that the strategies are aimed at very different under-served groups, it isn't possible to say which are most effective overall. E.g cultural competency would not be expected to increase participation of people with communication disabilities for whom easy read documents would be more effective.” | | |
| Please provide examples of what has worked particularly well, in which context (which under-represented group was included), and why: | | |
| “Online recruitment and follow-up particularly popular in ASCEND PLUS.”  “There is no clear evidence at the moment re beneficial strategies for EDI inclusion. We are therefore running various SWATs to contribute to this body of evidence.”  “When recruiting alcoholic's to a trail we found incentive payments were helpful in getting this hard to recruit population to come forward. In an obesity study offering of home visits for weighing individuals was offered as an option.”  “Translated PIS.”  “NA.”  “This has worked well in a social care study. In certain areas there were more participants who were unable to communicate in English and therefore would be excluded from the study. However, we had translators available which meant that they could participate.”  “It's difficult to know what has helped, I think the strategies have added very little.”  “Incentive payments. and videos.”  “During the COVID pandemic the ability to offer variation along the assessment pathway likely had a positive effect on recruitment / retention of participants. However, this may not be universal in nature given that each trial is specific and behavioural changes will likely occur in a post-pandemic phase.”  “Face to face (doorstep survey) targeting areas with higher ethic diversity, increased poverty and areas that usually do not respond to postal or online surveys.”  “Nil.”  “It is hard to know what effect any of the strategies we've implemented have had. I do feel that EDI in healthcare is a bigger issue than just trials - once we improve general healthcare engagement this should improve trials EDI.”  “GP texts to population we require advertising the study, which would include alot of south asians (weight management trial) and social media ads targeted to age, gender, regions required for our study.”  “Ensuring diverse PPI has helped to plan inclusive recruitment strategies, this wasn't an option listed above. Reimbursing travel has also been effective for those that couldn't otherwise afford it.”  “n/a”  “We have allowed sites to employ interpreting service at their hospital and feed the cost back to the study. This has resulted in some participant who would have otherwise not been included in the study participating.”  “We cannot be sure what has been successful really, but we have been using translation and this has led to the recruitment of people that would not have otherwise been included. PPI have advised us on including costs for participants, simplifying information and making videos but we do not know if this has led to recruiting people that would not have otherwise been recruited. recruiting outside of usual care pathways may have led to the recruitment of people who were not accessing healthcare, and usually not included in research for this reason.”  “Patient-specific videos in various languages that assist with the consent process have been particularly helpful.”  “NA”  “Infographics as a summary explanation for a number of trials to help patients’ engagement.”  "Again, hard to evidence my thoughts on this and this is perhaps an area we need more research on (and staff in our CTU are looking at this and trying to get funding for more research on what has worked). My opinion would be opening more sites in areas where more diverse communities access health care and using a range of methods to recruit into trials, and raise awareness, such as using social media."  “Multiple strategies, depending on the context. Some broader strategies include monitoring who is screened, approached, recruited (e.g SEAR framework), using more accessible and layered participant information, having flexible options for consent (e.g in person, online, by phone) and intervention delivery.”  “We are wanting to properly evaluate these processes in the near future.”  “We have not researched this yet.”  “Using the translation service and making it known that you are able to use the service and give exact instructions of when to use and how to use it. Often the problem is the cost of using the service as most trials are not funded for this.”  “Paying for any travel costs and reimbursing participants for their time.”  “Mobile phones in Africa, participants can keep the phones afterwards.”  “Don't know.”  “I work on old trials which aren't really focused on EDI.”  “Don't know yet.”  “Accessibility but at Trust level - they translate the information - we just ask them to.”  “Community champions work well in public health trials in underserved groups.”  “QR code for quick easy accessible information on the trial - ease of use, simple to implement, can be accessed anytime.”  “Difficult to tell at this stage as we are not yet seeing great improvements.”  “Giving parents/children a voucher helps engage the child in the visit. Guess that children have less of an understanding of doing research for potential benefits they won't see. Having something they get then and there makes them feel like they have helped and this is being appreciated.” | | |
| *Which of the following do you / people in your CTU cost for in your current grant applications (select all that apply)* | | |
| Not applicable (we don’t consider EDI strategies in any of our trials) | 2 (1.9%) | |
| Translated consent materials (e.g. consent form, PIL, information video) | 25 (23.4%) | |
| Translated questionnaires (paper and electronic) | 16 (15.0%) | |
| Interpreter services | 11 (10.3%) | |
| Extra time for staff to recruit and follow-up participants | 3 (2.8%) | |
| Engagement activities (e.g. costs to host an event with community groups) | 12 (11.2%) | |
| Translation of outputs (e.g. participant results leaflets or videos) | 13 (12.1%) | |
| Dissemination events | 13 (12.1%) | |
| Don’t know | 8 (7.5%) | |
| Other | 4 (3.7%) | |
| Comments, if Other:  “If recruitment is through NHS sites, then we tend to reflect on the needs of the trial and aim to plan for coverage of the above elements where applicable within adequate tariffs attribution in the SoECAT.”  “Will depend on the context, so not all are routinely costed in to all studies.”  “PPI meeting expenses.”  “Reimbursement for additional trial visits.” | | |
| *Thinking about your current trial(s), do you think the EDI budget adequately covers the activities required to recruit people from under-represented groups?* | | |
| Not applicable (we don’t consider EDI strategies in any of our trials) | 1 (3.0%) | |
| Definitely | 2 (5.0%) | |
| Very probably | 1 (3.0%) | |
| Possibly | 4 (11.0%) | |
| Probably not | 13 (34.0%) | |
| Definitely not | 11 (30.0%) | |
| Don’t know | 5 (14.0%) | |
| Comments:  “We are based in the South West and are local population is predominantly white caucasian, hence they tend to be the majority in our trials. However, there are areas of depravation in our area and these groups are often poorly represented in trials. There are a number of local schemes to try to improve this, for example, our local NHS Trust has a mobile research team equipped with vans that can be used as mobile clinics and take research out to these harder to reach groups. The University of Plymouth does have its own EDI policy although setting out the broad principles does not really cover clinical research. We tend to rely on the HRA guidance.”  “Yes, we planned for what we needed and the NIHR funded it.”  “We could have translated our documents as they are currently only available in English.”  “We have been costing for 5-7 languages, but this is not sufficient to cover all of the languages needed in the UK.”  “With the changes to NIHR's expectations, perhaps this will change for the better.”  “EDI components take a lot of time and effort to get right and apply and this has to run alongside setting up and managing the trials.”  “The trial staff may be tasked with running the EDI intervention, even if they have little or no experience in this field, so it would be good if funding was given for a specific person to manage the EDI components.”  “We have found document translation more expensive than planned, however use of interpreter services has not been as costly (due to lack of use) as we had planned for.”  “We don't have an EDI budget as such, we have been adding EDI related costs in our budget templates for trials over the last few years but will take some time to pull through due to set up times. We don't have any dedicated staffing for EDI so have been focusing on one thing at a time (starting with data collection/changes to budget templates).” | | |
| *Is anyone conducting research related to EDI (e.g. SWATs) in your CTU?* | | |
| Yes | 11 (30.0%) | |
| No | 12 (32.0%) | |
| Don’t know | 14 (38.0%) | |
| Please provide further details of the research project(s): | | |
| “Gender disparity in CV trials.”  “SWAT 145, SWAT 205.”  “We are aiming to integrate a monetary incentive-based SWAT into a large trial looking at post-mandible surgery antibiotic use. Following the award of an internal grant to look at SWATs priorities for the unit we aim to integrate SWATs into incoming trials we collaborate on.”  “An ongoing project through the Trial Forge SWAT Network and IMPLEMENT SWATs (NIHR Advanced Fellowship) has produced a list of priority SWATs which includes EDI SWATs. https://doi.org/10.17605/OSF.IO/CZ829.”  “One done a year or 2 ago I think.”  “I understand a statistician shared a poster on some research comparing two trials that had published at a recent ICTMC and is trying to develop a proposal to build on this.”  “There are SWATs and SWAPs e.g SWAPs exploring inclusion of people with MLTCs in SCC AFTER (radiotherapy trial) and TIPTOE (osteoarthirits self-management trial), and CONSULT SWAT (supporting consultees/legal representatives of people lacking capacity to consent) plus Talking Trials to engage people from ethnic minority and socio-economically disadvantaged communities in clinical trials.”  “CORAL study.”  “Include study starting about translation services.”  “Small project on language as a barrier to research inclusion. Ongoing SWAT to look at paper vs electronic PRO to see impact on retention (along with collecting demographics on who chooses what method).” | | |
| Thinking about the PPI group(s) for your current trial(s), how representative are its members (e.g. with regards to the demographic characteristics of the trial population) | | |
| The PPI group(s) is very representative | 6 (16.0%) | |
| The PPI group(s) is somewhat representative | 20 (54.0%) | |
| The PPI group(s) is not representative | 4 (11.0%) | |
| I don’t have a PPI group | 1 (3.0%) | |
| Don’t know | 6 (16.0%) | |
| Comments:  “It is hard to get representatives from poorer social groups to give up time to participate. PPI groups of patients do however, tend to be reasonably representative for gender, education and cultural background.”  “Some trials have more PPI contributors and, in these cases, there is better representation, but our PPI co-applicants are not very diverse.”  “I am not sure, and I don't believe we collect protected characteristics from them currently.”  “We tried very hard to get a representative PPI group but have ended up with a majority, white retired group.”  “Inclusion of those with neurodiversity and from the LGBTQ+ community.”  “We are working on this.” | | |

## Table S5. Description of communities involved in outreach

|  | **NIHR Leicester BRC** | | | | **Age UK** |
| --- | --- | --- | --- | --- | --- |
| **Community Partner** | Dance & Health [1, 2] | | | Green Pasture | Age UK Leicestershire & Rutland |
|  | Belgrave | Braunstone | Oadby |  |  |
| **Description of community** | Asian British Indian women, age range 60-80, average age 70. Lower English proficiency. IMD decile* 3. | White British, age range 40-90, average age 57. IMD decile 1. | Asian British Indian women, age range 40-80. IMD decile 9 | African-Caribbean church group, age range 20-70. IMD decile 3. | Community hub with ongoing activities (regular & drop-in), mixed background. Age range 65-80. IMD decile 3. |
| **Activity** | Dance &  discussion | | | Free drop-in games and activity | Discussion & Lunch |

* Note: IMD decile (according to English Index of Multiple Deprivation 2025) ranges from decile 1 (most deprived) to decile 9 (least deprived)

1.	Pritchard R, Darko N, Stevenson E. Enhancing community engagement, public involvement, and social capital through researchers’ participation in community dance projects: unexpected outcomes in underserved communities. Research Involvement and Engagement. 2024;10(1):79.

2.	Pritchard R, Bhavsar S, Campbell-Morris P, Modi P, Nugent M, Hughes J. Lessons from the field: The role of agility in a coproduction project encompassing the COVID-19 pandemic. Health Expectations. 2022;25(2):499-505.

## Table S6. Summary of barriers and facilitators identified, including evidence sources, which informed the first checklist draft

| **Process** | **Barrier** | **Facilitators / Strategies** | **Evidence Source** | | | |  |
| --- | --- | --- | --- | --- | --- | --- | --- |
|  |  |  | **Review** | **Community** | **Survey** | **Expert** | |
| Stakeholder engagement |  | **Consult with as many diverse demographics as possible** | **☒** | **☐** | **☐** | **☐** | |
|  |  | **Community-based participatory research principles (continued)**  • Development of community advisory board  • Community/Patient representative on trial / research steering group  • Co-design involving population of interest  • Feedback on study-related changes  • Stakeholder coordinator | **☒** | **☐** | **☐** | **☒** | |
| Representation | **Underrepresentation of racial and ethnic minorities among research personnel**  **Low numbers of minority investigators** | **Representative staff** (aka culturally congruent / concordant)  • Multilingual staff  • Community / citizen scientist  **Leadership & Development**  • Improve diversity and underrepresentation of clinical trial leadership and physicians  • Create programs that support minority and underrepresented investigators in research  • Recruitment and training of diverse healthcare providers and researchers (eg. apprenticeship program to create a pipeline of trained clinical research personnel) | **☒** | **☐** | **☒** | **☒** | |
| Lack of affinity with participants | **Lack of cultural awareness and sensitivity**  **Lack of knowledge and competence in dealing with minority groups** | **Researcher training**  • Cultural competence training for research staff: researchers given feedback on skills during recruitment  • Training on how to handle potential challenges  • Increase awareness of undocumented minority groups’ fears of working with government institutions  • Implicit bias training  • Trialists to undertake PPI training  • Training on inclusive communication methods (such as layering clinical trial information, participant centred communication, and providing guidance for recruitment of those with impaired capacity to consent)  • Careful consideration about how staff completes training (eg. those who need it are rarely people who attend training) | **☒** | **☐** | **☒** | **☒** | |
| Research question | **Research data not used to benefit community** | **Community-based participatory research principles**  Co-design involving population of interest starting from research question development | **☒** | **☒** | **☐** | **☐** | |
| Access to trial | **Lack of awareness of research opportunities**  **Lack of understanding about trial processes**  **Lack of knowledge on how to participate in a trial**  **Lack of access to clinical trials and its treatment**  • Lack of trial availability in community setting  • Minority population less likely to access primary healthcare services; more likely to access Emergency Services  • Receives healthcare at community centres with low trial enrolment rate | **Community outreach**  • Educational session for community  • Recruitment events  • Engagement via events and sessions (eg. “hub pop-ups”, market stalls)  • Explain relevance and benefits of research study to the community  **Advertisement (RO)**  • Digital engagement; advertise on publicly accessible platforms (eg. social media, online recruitment sites, university message boards, **NHS app**, email listservs to business and health service directories, Google Adwords system)  • Offline recruitment materials (eg. posters, business cards, newsletters) implemented in communities including: church lobbies, temples, mosques, (places of worship), recreational facilities, cultural events, festivals, corner store, pharmacy, postcard in daily newspaper, libraries, public housing resident meetings, barber shops, beauty shops, **supermarkets** and restaurants, shopping malls, stores in ethnic communities, YMCA, health ministries, **community centres**, health fairs, bus station, schools and colleges)  • Traditional offline methods (public transport advertisement, media advertisement including newspaper, TV, **radio** and billboards) in area of target population  • Word of mouth referrals  • Direct mail letters using GP patient lists, election list or geo-targeted lists, with prepaid opt-in or opt-out return cards  • Marketing strategies involving promotion and branding & logo  **Question prompt list for patients to ask clinicians about eligible clinical trials** | **☒** | **☒** | **☐** | **☒** | |
| Funding | **Lack of trial funding**  • Racial gap in trial funding  • EDI strategies not costed  • High startup cost for site (inclusive of resources required, research staff, time and training) | **Adequate budget at application stage**  • Undertake superficial equality impact assessment to inform funding application  • Calculate cost per patient to help move money between sites during a trial  • Include costs for video production and translation in funding applications  • Translation and interpreters to be factored into cost planning (Trial Forge Guidance 3 provides example costs)  • Cost consideration for alternative modes of trial delivery | **☒** | **☐** | **☒** | **☒** | |
|  | **No mandated diversity requirement in funded trials** | **Mandate diversity targets for funding**  **Diversity plan**  • Awareness of and familiarity with trial participant demographics  • Specific enrolment goal  • Monitoring of participant:prevalence ratio  • Appoint a diversity officer | **☒** | **☐** | **☒** | **☒** | |
| Ethics / legal or mandated actions |  | **Advocacy by ethics review boards**  • Implement requirements for researchers to collect and report sociodemographics and justify protocol design choices according to inclusion criteria  • Diversity and Inclusion Subcommittee within Research Boards | **☒** | **☐** | **☐** | **☐** | |
|  |  | **Legislation to improve diversity** (eg. NIH Revitalization Act of 1993, US FDA requiring demographic information prior to approval) | **☒** | **☐** | **☐** | **☐** | |
| Trial design | **Minimal consideration of social or political determinants of health in research design**  • Culturally insensitive research models due to lack of cultural humility  **Protocol missuited to accessibility requirements**  • Impaired health and comorbidities limit participation (eg. sensory or cognitive impairment, communication difficulties)  **Logistical difficulties within protocol (see also recruitment, logistical barriers)**  • High frequency of required attendance  • Time commitment and duration of study visits | **A needs analysis and flexibility in protocol to accommodate underserved groups and address barriers, including:** |  |  |  |  | |
|  |  | • Tailor study protocol to address identified barriers | **☒** | **☐** | **☒** | **☐** | |
|  |  | • Telemedicine visits | **☒** | **☐** | **☐** | **☒** | |
|  |  | • Options for partial trial participation | **☐** | **☒** | **☐** | **☐** | |
|  |  | • Offer services and referrals intended to address social inequalities (eg. gender-based violence, financial security) | **☒** | **☐** | **☐** | **☐** | |
|  |  | • Conduct research visits in individual setting | **☒** | **☐** | **☐** | **☐** | |
|  |  | • Conduct research visits conducted in group setting | **☐** | **☒** | **☐** | **☐** | |
|  |  | • Implement qualitative approaches to optimise recruitment (eg. Quintet Recruitment Intervention) | **☐** | **☐** | **☐** | **☒** | |
|  |  | **Conduct equality impact assessments** (eg. using the NIHR INCLUDE framework, Centre for Ethnic Health Research Equality Impact Assessment) **during trial design and development** | **☐** | **☐** | **☐** | **☒** | |
|  |  | **Identify which underserved populations would make up a purposeful study sample and recruit accordingly** | **☐** | **☐** | **☒** | **☒** | |
| Beliefs | **Different health priorities**  **Stigma**  • Stigma surrounding the condition of study  • Stigma about research participation  **Bias and under-appreciation of the prevalence of the disease condition**  **Lack of perceived benefits** (eg. perceived increased vulnerability to complications and side effects for certain disabilities; low expectation of therapeutic benefits)  **Perceived risk of increased workload on clinician** if patient deteriorates from trial  **Cultural differences** surrounding mental health, diet, body, help-seeking behaviours as examples  **Participant feeling they are not educated enough to take part or that they are unable to provide ‘good’ answers** | **Study material to align with participant motivation** (eg. altruistic motives, emotional appeal, prevention of disease including use of prevalence statistics)  (See also “inclusive study material”) | **☒** | **☒** | **☐** | **☒** | |
| Communication within research team |  | **Regular discussion with trial sites to understand if there are common barriers to recruitment** | **☐** | **☐** | **☐** | **☒** | |
| Eligibility criteria | **Unjustified and ambiguous research criteria**  Limiting eligibility criteria (direct or indirect)   - English language requirement - Ambiguous exclusion criteria - Exclusion of people with serious mental illness - Diagnoses not routinely recorded (learning disabilities) - Specific screening measures that might differ between communities (eg. pulse oximetry)   Additional legal requirements required (eg. prisoners or those with impaired capacity to consent) | **Non-discriminatory inclusion and exclusion criteria**  • Use of standardised tool rather than clinical judgement to reduce exclusion due to discriminatory biases  • Use of resources designed to help include those with impaired capacity to consent, such as the recommendations outlined on the CONSULT website | **☒** | **☒** | **☒** | **☒** | |
| Consent |  | **Safeguarding informed consent** (eg. address culturally relevant concerns during informed consent)  • Emphasis confidentiality  • Thorough explanation of study objectives and study risks and benefits to participants  • Provide counselling on side effects of experimental therapies | **☒** | **☒** | **☐** | **☐** | |
|  |  | **Shortened or simplified consent form**  **Flexible capacity assessment** (eg. a sliding scale of required capacity to ensure direct patient consent is considered as far as possible to accommodate limited comprehension) | **☒** | **☐** | **☐** | **☐** | |
| Language | **Language barrier**  • Low English proficiency  • No access to interpreter service or translated materials  • Researchers do not speak the same language as participants | **Translation and interpretation**  • Provision of interpreting and translation services  • Translated material in languages other than English  • Liaise with key voices in communities to advise on wording and translations  • Better translation standards  • If translation is used in recruitment materials, they should also be used during data collection and dissemination, and interpreters to be used throughout the duration of the trial as needed  • Identify processes of translation including back-translation and using interpreters  • Linkage with local authority interpreters, NHS interpretation services, or other professional translation/interpretation services  • Multilingual staff, see also “researcher” | **☒** | **☒** | **☒** | **☒** | |
| Delivery of information | **Poor quality communication**  • Use of jargon  • Information too long and dense*; legal requirements around fully informed consent and data protection (GDPR) determines the information required to be provided* (SU)  **“Over-communication”***  Emails from research teams on separate projects (same university) leading to feeling of overwhelm and resent; multiple interactions can feel predominantly transactional* (SU) | **Multiple communication strategies**  • Multiple modes of communication (website, phone, text, email, letter)  • Use of social media  • Patient centred and preferred communication method  • Text-to-speech technology for blind participants | **☒** | **☒** | **☐** | **☒** | |
|  | **Low literacy**  • Low educational attainment  • Low health literacy  • Low research literacy  • Lack of digital literacy | **Inclusive study material**  • Use of health literacy guideline (eg. avoid hyphenated word, use short words and action verbs)  • Material at low reading level (< 12 yrs)  • Accessible language or information: plain language without jargon, use of bullet points and large print, avoidance of negative terminology  • Visuals and text-enhancing images in study material  • Recruitment material to show a person of the race or ethnic group  • Multimedia use (eg. animations and videos)  • Use of culturally aware language (eg. using accepted terminology and cultural norms that communities find acceptable) ie. culturally tailored communication  • Accessible information for participants with intellectual disability  • (Co-produced) layers of information starting with most important to navigate complex information, to be provided in addition to legally required documentation | **☒** | **☒** | **☒** | **☒** | |
| Availability of information | **Lack of access to quality information; rampant misinformation** | **Widespread availability of information**  • Video-based education  • Education material created with patients, families and friends  • Availability of patient information in multiple languages to improve access | **☒** | **☒** | **☐** | **☒** | |
| Recruitment strategy | **Minority group is not the focus of recruitment strategy** | **Recruitment efforts and materials tailored to specific populations** (eg. language availability, advertisement in forum with which they have contact) | **☒** | **☒** | **☐** | **☐** | |
|  | **Numerous methods of recruitment can lead to more occurrences of human error, result in greater costs, and require more staffing resources** | **Multiple, diverse, and flexible recruitment strategies**  • Clinic-based in-person recruitment  • Innovative recruitment schemes (eg. open access screening programmes)  • Opt-out approach to recruitment  • Central patient registry of potentially eligible patients  • Local registry of interested individuals  • Using local community groups or external vendors, such as COUCH Health, Egality Health, and Health Watch, to identify diverse participants  See also “Access to trial, Advertisement” and “Community Outreach” | **☒** | **☒** | **☐** | **☒** | |
|  |  | **Site selection and diversification**  • Substudies or study arm in specific geographic area or centre to target minority population  • Encourage use of sites with high enrolment of underserved groups  • Increase number of recruitment sites  • Increase rural and community sites; sites in geographically underserved areas  • Venue-based sampling  • Close recruitment sites with low yield  • Decentralised trials (hub and spoke model using partnerships between large centres and satellite sites) | **☒** | **☐** | **☒** | **☒** | |
|  | **Limited clinician engagement with potential participant** | **Letters of support by community providers or physicians prior to study contact** | **☒** | **☒** | **☐** | **☒** | |
|  |  | **Collect data on reasons for participant drop out** | **☐** | **☐** | **☐** | **☒** | |
| Investigator bias and discriminatory sampling | **Attitudes and beliefs towards potential participants**  • Clinician concern about loss of trust from patient and family  • Preconceived notion that certain individuals would be poor candidates  • Clinician attitude about patient adherence  **Gatekeeping by carers, clinicians, and institution managers**  **Increased clinician burden** (eg. competing factors, limited time, knowledge, awareness of trials) | **Clinicians to offer trials to all eligible patients** | **☒** | **☐** | **☒** | **☐** | |
| Logistical factors | **Transport**  • Lack of transport  • Lack of parking  • Need to travel large distances to access trial | **Transport**  • Provide travel arrangements  • Free parking  • Flexible and accessible location including home visits, local clinics, GPs, and community venues  • Select locations accessible and trusted by community  • Mobile healthcare providers / units / clinical trials team  • Remote consent and screening | **☒** | **☒** | **☒** | **☒** | |
|  | **Finance**  • Inability to use financial incentives due to tax implications  • Lack of health insurance  • Un/underemployment | **Financial reimbursement, compensation, and incentive**  • Travel reimbursement  • Arrange travel costs upfront / Provide compensation at the time of research visits or asap  • Provide food / meal as compensation  • Non-financial incentives or “study swags”  • Lodging arrangement  • Alternative incentives (eg. certificates for children and letters that acknowledge participant contributions); co-create appropriate incentives where possible | **☒** | **☒** | **☒** | **☒** | |
|  | **Competing obligations**  • Childcare and other care responsibilities  • Work commitments  • Time commitment required and duration of study visits | **Competing obligations**  • Provide childcare / care  • Align study protocol with clinical visits to increase efficiency and fewer study visits | **☒** | **☒** | **☐** | **☐** | |
|  | **Researcher unavailable out-of-hours** | **Flexible timing**  • Extended office hours  • Out of hours contact | **☒** | **☒** | **☐** | **☒** | |
| Intervention | **Participant unable to choose preferred intervention** | [**Population-specific interventional mapping approach**](https://www.scopus.com/record/display.uri?eid=2-s2.0-85042164718&origin=inward) (based on health behaviour theories)  • Cultural adaptation of intervention (eg. including a trauma module in web-based learning intervention program) | **☒** | **☒** | **☐** | **☐** | |
| Follow-up | **Lack of follow-up** | **Regular and timely communication**, see also section on “Communication”  • Dedicated time and space for participants to ask questions  • Reminder calls or postcards  • Regular study updates  • Frequent check-ins (eg. weekly or monthly follow-up calls or sessions)  • Vigorous follow-up for missed appointments  • Study helpline & out of hours contact | **☒** | **☒** | **☐** | **☐** | |
| Data Collection & Analysis | **High prevalence of confounders in disabled groups** | **Report baseline characteristics** to allow stratification of results and statistical adjustment (subgroup analysis)  **Collect demographic data on participants but also those who refuse participation or are not being approached [although authors also recognised this data needs consent to be collected]**  **Incorporate sensitive assessment tools** that relate to the confounding factor rather than proxies (eg. Frailty index instead of age)  **Allow for proxy completion of data where possible (family members, friends, care workers or clinicians)** | **☒** | **☐** | **☒** | **☒** | |
| Dissemination | **Failure to disseminate information**  **Lack of outreach to communities** | **Increased transparency through process and report progress**  • Dissemination of research results to “close the loop” (eg. short videos or flyers circulated by local charities and community groups)  **Widespread availability of information**  See under “Availability of information” and “Community Outreach”  **Academic journals to require authors to report ethnicity data** | **☒** | **☒** | **☐** | **☒** | |
| Mistrust | **Mistrust of researchers**  **Mistrust in healthcare system**  **Awareness of history of unethical practices**  **• Historical trauma** | **Personalised approach to establish rapport**  • Self-disclosure by study staff to build relationships  • Engagement with individual participant to share values and connection to the community  • Show gratitude to patients  • Staff introduction using first name  • Allow participants the option to choose how they would like to be addressed  • Not use an overly critical language if participants are unable to attend or late  **Inclusion of pilot study results in PIL** | **☒** | **☒** | **☒** | **☒** | |
| Fear | **Fear of unintended outcomes and adverse effects**  **Fear of exploitation or being experimented on**  **Fear of deportation / immigration status**  **Fear of inadequate protection to ensure privacy**  **Lack of confidence to participate / anxiety** | **Thorough explanation of study objectives and study risks and benefits to participants**  (see also, Consent) |  | | | |  |
|  | **Lack of family and social support** | **Get information about research through family and friends**  **Social proof (testimonials or use of patient advocates)**  **Lay navigators / community liaison / research ambassador**  **Inclusion of family in research** | **☒** | **☒** | **☐** | **☒** | |
| Organisational guidance | **Lack of support and guidance from research bodies on inclusive practices** | **Dedicated consultation service offered to study team on enhancing community engagement and inclusion in research** | **☒** | **☐** | **☐** | **☐** | |
| Challenging social factors | **Structural inequities**  • Housing / homelessness  • Transport  • Poverty  • Access to healthcare (SU) | (See Logistical factors) | **☒** | **☐** | **☐** | **☒** | |
| Discrimination | **Systemic racism**  **Existing racial and ethnic inequities** |  | **☒** | **☒** | **☐** | **☐** | |
| Research infrastructure | **Inadequate research infrastructure**  **Lack of resources for health providers of disadvantaged groups**  **Inequities in referrals to clinical trial**  **Ethical restrictions barring direct contact with institutionalised groups** | **Community partnership**  • Work with organisations with whom longstanding trust relationships have been established  • Partnership with community leaders (eg. elders as family leaders and cultural brokers / church pastors for African American and Spanish population)  • Linkage with other researchers to identify others involved in community engagement activities and use their links to partner with community groups  • Training of community researchers  **Develop and strengthen research infrastructure**  • Local experts unaffiliated with trial  • Partner with local healthcare centres and practices  • Streamline referral system between community-based and academic centres  • Recurrent conversations with physicians and study staff about ongoing research  **Increase trial awareness** at hospital leadership level, by physician, and by community-based health providers  **Raise awareness of the need for innovative healthcare policies**  • Data sharing agreement approval including underserved group  • Leverage electronic databases to access diverse patient groups and to increase engagement and recruitment | **☒** | **☒** | **☒** | **☒** | |

## Table S7. Prototype Inclusivity Checklist (40-item) used at the Consensus Workshop

|  | **Checklist item** | **How** | **Yes** | **No** | **NA** |
| --- | --- | --- | --- | --- | --- |
|  | **Research priority setting** |  |  |  |  |
| 1 | Establish a community/patient advisory board to advise on all relevant aspects of your research portfolio and research priority setting | - Contact existing community partnerships, patient advocacy groups, charities, PPI networks at local, regional or national level - Contact the James Lind Alliance to see if unanswered research priorities/questions have been identified in your area of interest - Conduct priority setting exercise with research team and patient advocacy groups - Ensure mechanisms are in place to allow feedback of priority setting and milestones into the community (i.e. principles of Community Based Participatory Research) | ☐  ☐  ☐ | ☐  ☐  ☐ | ☐  ☐  ☐ |
|  | **Research investigator questions** |  |  |  |  |
| 2 | Use community/patient advisory board to refine the research question (patient population, interventions and outcomes) | - If not already established, see 1 above | ☐ | ☐ | ☐ |
| 3 | Characterise your patient population in terms of demographics (protected and other underserved characteristics) | - Check published literature, analysis of routine data / cardiovascular audits / data from trials, talk with clinicians treating the condition | ☐ | ☐ | ☐ |
| 4 | Define underserved characteristics you would like to target | e.g.   - People >80 years old, South Asian populations, socioeconomically deprived, etc. | ☐ | ☐ | ☐ |
| 5 | Identify patients/communities that represent those characteristics and recruit members to your patients advisory board / PPI group | - Contact your funding body to see if they offer a dedicated consultation on enhancing community engagement and inclusion in research - Refer to NIHR INCLUDE or Equality Impact Assessment available from the Leicester Centre for Ethnic Health - Recruit patients with underserved characteristics using clinicians/health professionals in healthcare/community settings - Diversify existing patient advisory groups / establish new advisory group - Invite members from the relevant community partnerships to be patient researchers - Establish dialogue early, ideally at the same time that you develop your research question | ☐  ☐  ☐  ☐  ☐  ☐ | ☐  ☐  ☐  ☐  ☐  ☐ | ☐  ☐  ☐  ☐  ☐  ☐ |
| a. | **EDI plan** |  |  |  |  |
| 6 | Undertake a “needs analysis” to identify the barriers (physical or otherwise) to participation in the research study and adapt the study design accordingly | - Present the research study to community groups, patient groups, patient advisory board, etc. to identify barriers to participation and potential solutions. This may require multiple meetings / iterations | ☐ | ☐ | ☐ |
| 7 | Include a dedicated EDI lead as part of the research team | - Recruit from your institution, community partnerships, etc. | ☐ | ☐ | ☐ |
| 8 | Ensure your research team are as representative of the underserved characteristics as possible |  | ☐ | ☐ | ☐ |
| 9 | Formulate an EDI plan (recruitment/retention intervention) and include in the study protocol | - Based on the needs analysis and including details on participant demographics, diversity target / enrolment goal, and how this will be monitored throughout the study - List recruitment intervention components and describe how these will be implemented | ☐  ☐ | ☐  ☐ | ☐  ☐ |
| 10 | Budget for EDI resources required (e.g. video production, translation and interpreters, community engagement activities, etc.) | - Check with funding body if they offer an EDI budget | ☐ | ☐ | ☐ |
| 11 | Explore the use of shared/central resources | - Consult your wider department, institution or other institutions to identify established PPI groups/connections or creative/production teams to aid with representational resources | ☐ | ☐ | ☐ |
| a. | **Study design** |  |  |  |  |
| 12 | Consider decentralised trials (move research delivery into communities) | - Conduct trial-related activities digitally, at homes of trial participants or at a facility which is local to trial participants | ☐ | ☐ | ☐ |
| 13 | Avoid discriminatory inclusion and exclusion criteria | e.g.   - Exclusion of people who don’t speak English or people who live far from the research site - The use of age as a proxy for frailty or multiple long term conditions (use Frailty index instead of age as an exclusion criterion) | ☐  ☐ | ☐  ☐ | ☐  ☐ |
| 14 | Ensure the protocol is flexible to accommodate the needs of underserved groups (based on needs analysis) | - Consider preference-based randomisation to allow inclusion of more underserved participants (if appropriate) - Consider elements of the protocol which could be optional, to allow for partial trial participation (if appropriate) - Consider making secondary or exploratory objectives optional (if appropriate) - Provide the option of remote/telemedicine research visits and procedures - Consider offering research procedures as both individual and group options, depending on participant preferences - Establish referrals to support services for participants who experience social inequalities - Consider embedding sub-studies in specific geographical areas to target underserved populations | ☐  ☐  ☐  ☐  ☐  ☐ | ☐  ☐  ☐  ☐  ☐  ☐ | ☐  ☐  ☐  ☐  ☐  ☐ |
| 15 | Consider setting recruitment targets for underserved groups |  | ☐ | ☐ | ☐ |
| a. | **Study delivery** |  |  |  |  |
| b. | *Recruitment and retention strategies* |  |  |  |  |
| 16 | Provide education on, raise awareness of, and promote interest in the study | e.g.   - Through community engagement officers, community partnerships, community advocacy groups - Marketing strategies involving promotion and branding | ☐  ☐ | ☐  ☐ | ☐  ☐ |
| 17 | Ensure recruitment strategies are multiple, diverse, and flexible | e.g.   - Patient registries, health service directories, direct mail letters using GP lists, word of mouth referrals, local community groups, external vendors (to advertise research study as widely as possible) - Implement offline recruitment strategies in locations relevant to populations of interest (places of worship, supermarkets, and community centres) - Implement online recruitment through digital platforms such as social media, NHS app, and forums with which populations of interest have contact | ☐  ☐  ☐ | ☐  ☐  ☐ | ☐  ☐  ☐ |
| 18 | Ensure mechanism is in place for feedback on recruitment method | - Discuss recruitment process with community advisory group and/or patient population and optimise accordingly | ☐ | ☐ | ☐ |
| 19 | Develop a communication plan within the study to ensure regular and timely communication with recruiting sites on the topic of representative recruitment | - Provide recruitment by underserved characteristics at Trial Management Group (TMG) meetings and in TMG reports | ☐ | ☐ | ☐ |
| 20 | Include research sites from geographically underserved areas | - Identify neighbourhoods/postcodes with high socioeconomic deprivation using Indices of Multiple Deprivation/Lower-layer Super Output Area Level and target recruitment of sites at these postcodes - Use UK Census data to identify population demographics (e.g. health, religion, ethnic group, economic activity, household deprivation, etc.) in different areas of the UK | ☐  ☐ | ☐  ☐ | ☐  ☐ |
| 21 | Ensure research staff have received training in cultural competence, implicit bias, and inclusive communication methods | e.g.   - Leicester Centre for Ethnic Health Cultural Competency Training course | ☐ | ☐ | ☐ |
| 22 | Ensure you have clinician/healthcare professional for recruiting underserved populations | - Communicate EDI plan with site PIs - If possible, recruit PIs representing underserved characteristics (e.g. women, people from an ethnic minority) | ☐  ☐ | ☐  ☐ | ☐  ☐ |
| 23 | Consider how clinicians will support recruitment of under-served groups | e.g.   - Endorse study through consultations, letters of support | ☐ | ☐ | ☐ |
| a. | *Patient facing materials* |  |  |  |  |
| 24 | Ensure patient information leaflets (PIL) and other study materials are written in easy-read English | - Use health literacy guidelines /plain English guidance to write patient facing materials - Co-produce study material with individuals from the underserved groups / community advisory boards, tailoring study materials using culturally accepted terminology - Adapt study material to include visuals and text-enhancing images. Where possible, the images should reflect the population of interest (e.g. diverse ethnicity) - Incorporate multimedia use such as animations and videos (or include links to online sources for these) for people with low literacy (either in English or other languages) - Explore feelings of relevant community / patient groups, and address these specifically in research materials | ☐  ☐  ☐  ☐  ☐ | ☐  ☐  ☐  ☐  ☐ | ☐  ☐  ☐  ☐  ☐ |
| 25 | Include pilot or feasibility study result in PIL where possible |  | ☐ | ☐ | ☐ |
| 26 | Shorten or simplify consent forms, where possible |  | ☐ | ☐ | ☐ |
| 27 | Consider scenarios of impaired capacity to consent during trial participation and establish assent procedures | - Assent options, questionnaire completing with help from others |  |  |  |
| a. | *Translation / interpreting* |  |  |  |  |
| 28 | Translate materials in other languages, as required | - Budget for translation / interpreting services in commonly-spoken languages - Use post-translation review with people who speak the language/s to check understanding - Consider using Artificial Intelligence (AI) translations (text, verbal and/or video) in recruitment pathways when there is no time to translate patient material (e.g. to introduce a study) | ☐  ☐  ☐ | ☐  ☐  ☐ | ☐  ☐  ☐ |
| 29 | Ensure access to communication throughout the research for people who do not speak English | - Employ multilingual staff or identify staff who can communicate in the native language with the population of interest | ☐ | ☐ | ☐ |
| a. | *Logistics* |  |  |  |  |
| 30 | Provide study participants compensation for their time and/or other incentives | - Reimbursement may be monetary or vouchers - Consider provision of snacks/meals if research visits are prolonged | ☐  ☐ | ☐  ☐ | ☐  ☐ |
| 31 | Discuss options for assisting study participants who are carers | e.g.   - Provide reimbursement for care (e.g. childcare or other care responsibilities) | ☐ | ☐ | ☐ |
| 32 | Provide accessible and flexible study locations and/or transport for research visits | - This can include home visits, local clinics, GPs, or community venues - Transport options include free parking, transport (e.g. taxi), or reimbursement of transport cost | ☐  ☐ | ☐  ☐ | ☐  ☐ |
| 33 | Extend office hours and ensure contact is available out of hours |  | ☐ | ☐ | ☐ |
| a. | *Data collection* |  |  |  |  |
| 34 |  | - Collect demographic data on participants - Collect anonymised demographic data on eligible population who refuse participation or are not being approached - Collect data on reasons for participant drop out - Allow for proxy completion of data where possible (family members, friends, care workers or clinicians) | ☐  ☐  ☐  ☐ | ☐  ☐  ☐  ☐ | ☐  ☐  ☐  ☐ |
| 35 | *Communication* |  |  |  |  |
| 36 | Outline communication strategies throughout the research process, offering multiple methods based on participants’ preferred communication method | - Capture method for preferred communication method in consent form (e.g. website, phone, text, email, post) |  |  |  |
| 37 | Establish rapport with study participants using a personalised approach | e.g.   - Showing gratitude to patients - Staff introduction using first name - Allowing participants the option to choose how they would like to be addressed - Staff self-disclosure (where applicable) to build relationship - Dedicated time and space for participants to ask questions - Reminder calls or postcards - Regular study updates - Frequent check-ins (e.g. weekly or monthly follow-up calls or sessions) - Vigorous follow-up for missed appointments - Study helpline & out of hours contact | ☐  ☐  ☐  ☐  ☐  ☐  ☐  ☐ | ☐  ☐  ☐  ☐  ☐  ☐  ☐  ☐ | ☐  ☐  ☐  ☐  ☐  ☐  ☐  ☐ |
| 38 | With permission from study participants, include family members as much as possible throughout the research process |  | ☐ | ☐ | ☐ |
| a. | **Study reporting** |  |  |  |  |
| 39 | Report baseline characteristics by underserved groups to allow stratification of results and potential for subgroup analysis |  | ☐ | ☐ | ☐ |
| a. | **Impact, dissemination and engagement** |  |  |  |  |
| 40 | Ensure dissemination of research results to participants and wider relevant communities, in collaboration with patient representative/EDI lead | e.g.   - Short videos or flyers circulated by/within local charities, community groups, and patient population | ☐ | ☐ | ☐ |

## Table S8. Consensus workshop breakout group sessions

**Breakout group session 1**: ***Review of current checklist***

In this session, please review your allocated section of the Inclusivity Checklist (draft version 0.5) and consider the following:

- Are there any strategies that are missing?
- Do you agree with the existing strategies?
- Is the language clear and unambiguous?
- If not, how should it be worded?

**Breakout group session 2**: ***Case studies and good practice***

In this session, please consider whether there are any examples or case studies you are aware of that demonstrate good practice of inclusivity in research. Thinking specifically about each checklist item, ***how*** can and should it be achieved?

**Breakout group session 3: *Defining the minimum criteria***

In this session, please discuss which checklist item(s) should be the minimum criteria. In other words, what is the minimum that all researchers should do, and what are optional items that researchers should consider?

## Table S9. Equality, Diversity and Inclusion Group of the National Cardiac Surgery Clinical Trials Initiative (Group Authorship)

| **Name** | **Affiliation** |
| --- | --- |
| Enoch Akowuah | South Tees NHS Foundation Trust |
| Allyson Arnold | British Heart Foundation Clinical Research Collaborative |
| Joanna Bell | NIHR Leicester Biomedical Research Centre |
| Judith Bower | National Cardiac Surgery PPI Group |
| Laura Green | Queen Mary University of London and NHS Blood and Transplant |
| Hannah Gilbert | University of Leicester |
| Emma Hopkins | Bristol Heart Institute |
| Rebecca Maier | South Tees NHS Foundation Trust |
| Ayodeji Ogunbuyide | NIHR Leicester Biomedical Research Centre |
| Mariana Rodas | British Heart Foundation Clinical Research Collaborative |
| Julie Sanders | King’s College London; The Women in Cardiovascular Trials (WiCVT) Group |
| John Maltby | University of Leicester |
| Hannah Gilbert | University of Leicester |
| Andrew A Klein | Royal Papworth Hospital, Cambridge |
| Riccardo Abbasciano | University of Leicester |

# Table S10. Inclusivity Checklist with full guidance

## Inclusivity Checklist for Clinical Research

|  | **Checklist item** | **Yes** | **No** | **NA** |
| --- | --- | --- | --- | --- |
| **Setting research priority and research question** | |  |  |  |
| 1 | Describe the diversity of the target population. |  |  |  |
| 2 | Engage with under-represented communities relevant to the target condition. |  |  |  |
| 3 | Include members of the community as research partners. |  |  |  |
| 4 | Evidence activities that raise awareness of and promote interest in the study amongst under-represented communities. |  |  |  |
| **Research Inclusion considerations** | |  |  |  |
| 5 | Describe the Research Inclusion Plan in the study protocol. |  |  |  |
| 6 | Budget for the Research Inclusion Plan in the funding application. |  |  |  |
| 7 | Designate individual(s) responsible for delivering the Research Inclusion Plan. |  |  |  |
| 8 | Document diversity of the research team. |  |  |  |
| 9 | Embed an evaluation of the Research Inclusion Plan in the study. *(optional)* |  |  |  |
| **Study design** | |  |  |  |
| 10 | Describe how inclusion and exclusion criteria mitigate discriminatory bias. |  |  |  |
| 11 | Document how protocol flexibility accommodates the needs of under-represented groups. |  |  |  |
| 12 | Pre-specify representative recruitment targets, ensuring these are reviewed throughout the research. |  |  |  |
| 13 | Document the use of decentralised trial methods (move research delivery into communities) or non-traditional trial designs. *(optional)* |  |  |  |
| 14 | Include options for partial participation, assent, and permission for future study contact in the consent form. *(optional)* |  |  |  |
| **Study delivery** | |  |  |  |
| *Recruitment and retention strategies* | |  |  |  |
| 15 | Document how recruitment strategies are multiple, diverse, and flexible to address inclusivity. |  |  |  |
| 16 | Specify mechanisms of feedback about recruitment and retention with recruiting sites and under-represented groups. |  |  |  |
| 17 | Document plan for inclusion of research sites from geographically under-represented areas. |  |  |  |
| 18 | Document that research staff have received training in cultural competence, implicit bias, and inclusive communication. |  |  |  |
| 19 | Document how people with lived experience of the target condition, clinicians, and healthcare professionals have helped with recruitment of under-represented groups. |  |  |  |
| 20 | Document strategies used to increase accessibility for participants from under-represented communities (e.g. compensation, logistics, and travel arrangements). |  |  |  |
| *Communication Strategy* | |  |  |  |
| 21 | Document steps to ensure study materials such as Patient Information Leaflets and Consent Forms are short, simple, and written in easy-to-read language. |  |  |  |
| 22 | Document the strategies available throughout all stages of the research for people with communication difficulties (e.g. do not speak English, sight or hearing impairments). |  |  |  |
| 23 | Document how communication strategies reflect participant preference. |  |  |  |
| **Data Collection and Reporting** | |  |  |  |
| 24 | Collect comprehensive demographic data on participants. |  |  |  |
| 25 | Report results stratified by under-represented characteristics or sub-groups. |  |  |  |
| **Impact, dissemination and engagement** | |  |  |  |
| 26 | Document dissemination of research results to participants and wider relevant communities. |  |  |  |

*We would like to acknowledge everyone who has contributed their expertise and time in the production of the Inclusivity Checklist.*

*We would especially like to thank our patient, community and public partners who have steered and shaped this project.*

## Glossary of terms

| **Term** | **Other terms used in literature** | **Definition** |
| --- | --- | --- |
| **Under-represented** | Under-served | These terms are often used interchangeably in health research. However, under-served generally refers to groups of people that don't have equal access to healthcare and research-based treatments and consequently experience poorer health outcomes. Under-represented refers to groups of people that are less represented or included in research that one would expect from population estimates. |
| **Research inclusion** | Equality, Diversity, and Inclusion (EDI) in research | The practice of including and accommodating people who may face discrimination and exclusion, either directly or indirectly, due to characteristics such as the protected characteristics under The Equality Act 2010. Research inclusion ensures fair opportunity for all to participate in research. |
| **Community Researcher** | Community officers  Community brokers  Community liaison  Research link workers  Citizen scientist  Lay navigators  Research champions  Health ambassadors  Community champions  Community engagement officer  Public engagement coordinator | A Community Researcher is a paid employment role as part of the research team. They are members from under-represented communities, and provide linkage of the research project to the communities. They may be employed on per project basis, although their role may be more effective in a long-term collaboration. Examples include Health Research Ambassadors that work with [NIHR ARC West](https://arc-w.nihr.ac.uk/research/projects/health-research-ambassadors/) and [Caafi Health](https://www.caafihealth.org.uk/our-health-ambassadors). |
| **Community Engagement Officer** |  | More commonly found in organisations such as the NIHR, Community Engagement Officers represent the organisation and establish linkage with local communities. They may or may not be a member of under-represented communities. This is a paid employment role as part of the organisation. |
| **Research Champions** |  | Research champions are individuals who undertake a role to promote and advocate research *generally* to other patients and members of the public; this is often done by individuals with the lived experience on a voluntary basis (with PPIE payment) and examples include [NIHR Research Champions](https://www.nihr.ac.uk/get-involved/spread-the-word). |
| **Study / Trial Management Group** | Research team | Group responsible for research (from design, delivery to dissemination); ideally consist of stakeholders including researchers, clinical experts, patient and public representatives, and a Research Inclusion Lead. |
| **Research Inclusion Lead** | Diversity officer  Research inclusion specialist | A member of the Study / Management Group who will have a primary role in drafting the Research Inclusion Plan, engaging with communities, keeping a log of activities, presenting research inclusion on agenda items in regular research meetings, monitoring inclusion progress, and providing feedback and updates to the communities. |
| **Representative** |  | In the context of research participation, the characteristics of interest in the research population is proportional to what one would expect from population estimates. |

## Guidance on the Inclusivity Checklist

|  | **Checklist item** | **How** |
| --- | --- | --- |
|  | Setting research priority and research question | |
| 1 | Describe the diversity of the target population.  *Demographic characteristics may include age, gender, ethnicity, socioeconomic status, disability, and other under-represented characteristics* | • Check published literature, including clinical trials data, cardiovascular audits / registries, and data collected as part of routine care.  • Speak with healthcare professionals treating the conditions about the characteristics of the people they treat.  • Undertake preliminary scoping analyses of disease epidemiology where these have not been performed. |
| 2 | Engage with under-represented communities relevant to the target condition. | • Ideally, establish the research questions *with* the communities. What are important questions that the communities want answered?  • Explore existing community partnerships, patient advocacy groups, patient support groups, charities, and PPI networks at local, regional or national level. See Appendix 1 for a list of existing PPI groups within cardiovascular research.  • Link with other researchers involved in community engagement activities and use their links to partner with community groups.  • Case Study 1: NIHR Leicester BRC Dance & Health Project  • If no known community partnerships exist, identify community leaders and begin building partnerships. This may involve speaking with local religious leaders, organisers of regular community meet-ups, public relations officers at charitable organisations, or patient and community engagement officers within institutions or organisations. |
| 3 | Include members of the community as research partners.  *This may be either as a patient or public representative on the trial/study management group or a “Community Researcher” involved with the local research team.* | • Highlight to the community the significance of their involvement in health research and invite them to take part in steering the research. Resources such as a spoken word film [‘~~IN~~VISIBLE’](https://egality.health/be-visible) may be useful to share.  • Ensure clearly defined Terms of Reference for Patient and Public Involvement (PPI), or job specification for Community Researchers. See Appendix 2 for examples.  • Provide adequate training to research partners. This includes training for PPI or research delivery such as facilitation skills.  • Payment should be provided for PPIE as per [NIHR guidelines](https://www.nihr.ac.uk/payment-guidance-researchers-and-professionals); Community Researchers as per institutional employment policy.  • Case Study 2: Deep End Research Alliance (DERA) and Community Research Link Workers. DERA also provides a useful [Community Researcher Toolkit](https://sites.google.com/sheffield.ac.uk/dera/research-within-communities/community-researcher-toolkit) for consideration.  • Diversify existing PPIE groups or establish new PPIE group.  • Ensure mechanisms are in place to allow feedback to the community regarding priority setting and milestones throughout the research cycle, using principles of Community Based Participatory Research (CBPR).^1.2^ CBPR is an equitable and collaborative research approach involving community members, researchers, and stakeholders. |
| 4 | Evidence activities that raise awareness of and promote interest in the study amongst under-represented communities. | • Undertake community outreach and educational events to build relationship and promote trust.  • Engage with local communities by attending local events or festivals and celebrations. For example, having a ‘market stall’ to share health-related knowledge and promote research study at monthly events within the local community centre.  • Implement marketing strategies involving promotion and branding. For example, using a study-specific logo on all participant facing material. |
|  | Research Inclusion considerations | |
| 5 | Describe the Research Inclusion Plan in the study protocol. | *This is now mandatory for all NIHR submissions.*  • Present the study to community groups and relevant PPI to identify barriers to participation and potential solutions. For example, mobile apps may be challenging for people who are older with lower digital literacy; paper version alternatives should be provided instead.  • Refer to existing guidance on EDI plan. Some resources are included here:   - [NIHR INCLUDE](https://www.nihr.ac.uk/improving-inclusion-under-served-groups-clinical-research-guidance-include-project). The NIHR-INCLUDE Project and Trial Forge have multiple [frameworks](https://sites.google.com/nihr.ac.uk/include/home?pli=1#h.rmza8jtu0kzi) including ethnicity, impaired capacity to consent, and socioeconomic disadvantage. - [Leicester Centre for Ethnic Health Equality Impact Assessment](https://arc-em.nihr.ac.uk/arc-store-resources/equality-impact-assessment-eqia-toolkit?node=1027&file=Toolkit&file_id=0) - [Leicester Centre for Ethnic Health Toolkit for Increasing the Participation of Rural and Coastal Communities in Health and Social Care Research](https://ethnichealthresearch.org.uk/research/resources-2/increasing-diversity-in-research-2/toolkit-for-increasing-theparticipation-of-rural-and-coastal-communities-in-health-and-social-care-research/) - [FOR Equity web-based tools and resources to reduce social and health inequalities](https://forequity.uk/) - [HRA/MHRA Inclusion and Diversity Guidance](https://www.hra.nhs.uk/about-us/news-updates/hra-mhra-guidance-developing-and-submitting-inclusion-and-diversity-plan/) [currently under consultation] - [NIHR Research Inclusion Toolkits Hub](https://learn.nihr.ac.uk/course/view.php?id=1215)   • Case study 3: Research Inclusion Plan from the CORAL study (NIHR152257) |
| 6 | Budget for the Research Inclusion Plan in the funding application. | • Contact your funding body to see if they offer a dedicated consultation on enhancing community engagement and inclusion in research.  • Consider including costs for a Research Inclusion Lead (see 7 below).  • Budget for Community Researchers, training resources and upskilling of research team, interpreting & translation services, alternative communication resources including visual aids, videos and animations.  • Consult your wider department and institutions locally or nationally to identify established resources to improve inclusion.  • See Appendix 3 for further considerations in costing inclusive research. |
| 7 | Designate individual(s) responsible for delivering the Research Inclusion Plan. | • Appoint a Research Inclusion Lead if resources permit.  • Alternatively, designate a member of the Study/Trial Management Group with knowledge and interest on research inclusion and diversity.  • If not possible, have the Research Inclusion Plan as a permanent review item on the agenda of research meetings to ensure its implementation. |
| 8 | Document diversity of the research team. | • Consider [‘positive action’](https://www.gov.uk/government/publications/positive-action-in-the-workplace-guidance-for-employers/positive-action-in-the-workplace#protected-characteristics) measures when hiring new researchers.  • Extend the research team to include Community Researchers.  • If possible, principal investigators (PI) at recruitment sites should reflect under-represented characteristics (e.g. women, people from an ethnic minority) |
| 9 | Embed an evaluation of the Research Inclusion Plan in the study. *(optional)* | Methods of evaluation include:  • Keeping a log of activities related to the Research Inclusion Plan (who implements each component and how)  • Collecting information on use of specific components of the research plan (e.g. how often are interpreting services and translated study materials used, how many participants are recruited through Community Researchers or social media campaigns)  • Questionnaires to recruiters and participants about acceptability and usability of specific components of the inclusion plan |
|  | Study design | |
| 10 | Describe how inclusion and exclusion criteria mitigate discriminatory bias. | • Consider whether the proposed criteria directly or indirectly discriminate against groups of individuals. For example, specifying that people should be “able to read and understand English” excludes people who do not speak English well or at all. Participant use of digital technology (e.g. mobile phone app) excludes those without phone or internet access or have lower digital literacy.  • Use validated measures in inclusion and exclusion criteria rather than proxy measures. For example, the Frailty Index should be used instead of an age cut-off in frailty-related studies. |
| 11 | Document how protocol flexibility accommodates the needs of under-represented groups. | • Consider preference-based randomisation to allow inclusion of more under-represented participants (if appropriate).  • Consider elements of the protocol which could be optional, to allow for partial trial participation (if appropriate).  • Consider making secondary or exploratory objectives optional (if appropriate).  • Consider offering research procedures as both individual and group options, depending on participant preferences.  • Consider reasonable adjustments that may be required for a participant to complete a study (e.g. having carer or family support to attend appointments, budgeting double time for trial activities where interpreter is required).  • Establish referrals to support services for participants who experience social inequalities. |
| 12 | Pre-specify representative recruitment targets, ensuring these are reviewed throughout the research. | • Recruitment targets should be set through discussions between the research team, under-represented communities, and other relevant stakeholders.  • Targets should be reviewed regularly throughout and after the research, and the results shared with all stakeholders including funders providing an EDI budget. |
| 13 | Document the use of decentralised trial methods (move research delivery into communities) or non-traditional trial designs. *(optional)* | • Consider conducting trial-related activities digitally, at homes of trial participants or at a healthcare facilities local to trial participants where possible.  • Provide the option of remote or telemedicine research visits and procedures  • NIHR provides further guidance and case studies on decentralised trials [here](https://sites.google.com/nihr.ac.uk/remotemethodsoftrialdelivery/)  • HRA also provides links to key resources [here](https://www.hra.nhs.uk/planning-and-improving-research/policies-standards-legislation/clinical-trials-investigational-medicinal-products-ctimps/decentralised-trial-methods-position-statement/) (see section on Links to key resources)  • Consider non-traditional trial designs, if appropriate, such as the hub and spoke model or a nested trial design  • Case study 4: BCIS4, an RCT utilising the hub and spoke model for delivery  • Case study 5 ROMA-WOMEN, a nested trial dedicated to women |
| 14 | Include options for partial participation, assent, and permission for future study contact in the consent form. *(optional)* | • Assent options, such as questionnaire completion with help from others, should be considered in scenarios of impaired capacity during trial participation.  • If appropriate, include options for partial participation. For example, if collecting multiple tissue samples such as blood, saliva, and urine, allow participants the choice of samples to donate.  • Include other optional clauses around contact about other research studies in the future. For example, “I agree to being contacted with details of future relevant research and to my contact details being stored at [location] for this purpose.” |
|  | Study delivery | |
|  | ***Recruitment and retention strategies*** | |
| 15 | Document how recruitment strategies are multiple, diverse, and flexible to address inclusivity. | • Depending on the context of the study, utilise any of the following:   - Advertise the research study as widely as possible using patient or research registries, health service directories, direct mail letters using GP lists, word of mouth referrals, local community groups, NIHR Patient Recruitment Centres. and external vendors. A small list of known external vendors is available in Appendix 4. - Implement offline recruitment strategies in locations relevant to populations of interest. For examples, places of worship, supermarkets, pharmacies, public libraries, local events, barber shops, and community centres. Other methods may include public transport advertisement, media advertisement such as specific radio stations, or billboards in areas where the target population lives. - Implement online recruitment through digital platforms such as social media, NHS app, online forums, and media sources which is accepted by the populations of interest.   • Consider the [QuinteT Recruitment Intervention (QRI)](https://www.bristol.ac.uk/population-health-sciences/research/groups/social-sciences-health/quintet/the-integrated-qri-for-rcts-that-anticipate-recruitment-challenges/) or similar qualitative research embedded within trials to optimise recruitment. |
| 16 | Specify mechanisms of feedback about recruitment and retention with recruiting sites and under-represented groups. | • This should detail mechanisms for discussion around recruitment and retention with:   - Recruiting sites; regular communication to identify barriers to recruitment - Under-represented communities involved with study; to continue engagement and optimise recruitment and retention. This discussion should start as early as possible, ideally at the study design stage. - Study participants; see item 23.   • Provide and discuss recruitment progress by under-represented characteristics at Trial / Study Management Group meetings. |
| 17 | Document plan for inclusion of research sites from geographically under-represented areas. | • Identify neighbourhoods or postcodes with high socioeconomic deprivation using Indices of Multiple Deprivation/ Lower-layer Super Output Area Level and target recruitment of sites at these areas.  • Use UK Census data to identify population demographics (e.g. health, religion, ethnic group, economic activity, household deprivation, etc.) in different areas of the UK.  • Consider embedding sub-studies in specific geographical areas to target under-represented populations. |
| 18 | Document that research staff have received training in cultural competence, implicit bias, and inclusive communication. | • Mandate inclusion training, similar to mandated Good Clinical Practice (GCP), for all members of the research team.  • Available training modules:   - *NIHR Learn modules* (free), including:   - Introduction to cultural sensitivity in research   - Inclusive language and communications guide   - Increasing participation of ethnic minorities in health and social care research   - EDI in Health and Social Care Research   - Research involving participants lacking mental capacity - *Leicester Centre for Ethnic Health* [*Cultural Competency Training*](https://ethnichealthresearch.org.uk/training/cultural-competence/) (£400; 50% discount for public sector organisations)   • Use the above resources and your community partnerships / patient advisory groups to create a bespoke training plan that meets the needs of your study  • Dedicate time during all Site Initiative Visits (SIV), if applicable, to research inclusion. This should at the minimum address the Research Inclusion Plan of the specific study, training on importance of diversity, resources dedicated to the specific study such as translation, interpretation, and budget for longer visits. |
| 19 | Document how people with lived experience of the target condition, clinicians, and healthcare professionals have helped with recruitment of under-represented groups. | • Ask recruiters (for example, clinicians and healthcare professionals) to target recruitment of study participants with under-represented characteristics.  • Provide endorsement by patients with lived experience, such as weblinks to [patient experience videos](https://www.youtube.com/channel/UCzhYWhS8XNXNFO-jb3o1HVQ) on the participant recruitment flyer or participant information leaflet; or by clinicians demonstrating support for research participation during clinical consultations or via letters of support. |
| 20 | Document strategies used to increase accessibility for participants from under-represented communities (eg. compensation, logistics, and travel arrangements). | Consider providing:  • Compensation for time and expenses (reimbursement may be monetary or vouchers).  • Reimbursement for care (e.g. childcare or other care responsibilities).  • Provide snacks or meals if research visits are prolonged.  • Accessible and flexible study locations, such as home visits, local clinics, GPs, or community venues  • Transport options, including free parking, transport (e.g. taxi) or reimbursement of transport cost.  • Extended office hours and or ensure contact is available out of hours.  • Align study protocol with clinical visits to increase efficiency and fewer study visits. |
|  | ***Communication Strategy*** | |
| 21 | Document steps to ensure study materials such as Patient Information Leaflets and Consent Forms are short, simple, and written in easy-to-read language. | • Use health literacy guidelines and plain English to write participant facing materials.  • Use readability tools such as FOG index to assess participant facing materials.  • Remove *all* jargon from participant facing materials.  • Provide a top-page concise overview of the research study on the Participant Information Leaflet (PIL).  • Co-produce study material with individuals from under-represented communities.  • Include visuals and text-enhancing images. The images used should reflect the population of interest (e.g. ethnic diversity, disability) if possible.  • Include pilot or feasibility study result in PIL where available.  • Culturally adapt the materials where possible. This may include tailoring study materials using accepted terminology or exploring feelings of relevant communities and addressing these in the materials.  • Case study 6: the ADAPTABLE study, an example of culturally tailored recruitment material  • Incorporate multimedia use such as animations and videos for people with low literacy.  • User language to appeal to people’s motivation, e.g. use phrases such as “Can you help?” to appeal to a potential participant’s altruistic motives.  • For complex materials, layer information starting with the most important message.  • Consider design aesthetics of materials, including images, colour, font style, size, and branding.  • See Appendix 5 for additional resources for creating participant facing material including readability checkers. |
| 22 | Document the strategies available throughout all stages of the research for people with communication difficulties (e.g. do not speak English, sight or hearing impairments). | • Note that communication difficulties encompass a large range of distinct challenges. For example, language barrier is different from hearing impairment, requiring different strategies.  • Budget for translation (forward and backward translation) of participant facing materials and interpreting services including but not limited to commonly spoken languages, British Sign Language, and braille.  • If materials are translated, ensure post-translation review is undertaken by both professional agency as well as individuals from the communities. A post-translation review will check for errors and ensure the translation reads correctly especially after formatting; this is particularly important for languages that are not written left-to-right like English.  • Explore the use of digital translation platforms (e.g. Pocketalk, DeepL, Google Translate) to introduce a study to potential participants who do not speak English.  • Case study 7: Pocketalk  • Employ multilingual staff or identify staff who can communicate in the native language with the population of interest.  • With permission from study participants, include family members as much as possible throughout the research process. |
| 23 | Document how communication strategies reflect participant preference. | • Make available multiple modes of communication such as email, telephone, video call, or post.  • Capture the preferred communication method for each participant.  • Have a dedicated study helpline or central email account that all participants can use.  • Provide reminder calls or postcards for study visits and ensure vigorous follow-up for missed appointments  • Provide regular study updates and frequent check-ins with participants (e.g. weekly or monthly follow-up calls or sessions)  • Establish rapport with study participants using a personalised approach. Examples include:   - Showing gratitude to participants - Staff introduction using first name - Allowing participants the option to choose how they would like to be addressed - Staff self-disclosure (where applicable) to build relationship - Dedicated time and space for participants to ask questions - Birthday and thank-you cards |
|  | Data Collection and Report |  |
| 24 | Collect comprehensive demographic data on participants. | • Collect demographics as part of study data with ethical approval. This may require explicitly asking participants their ethnicity or other characteristics as these are often poorly documented in medical records. It is also important to ensure and reiterate transparency to participants as to why specific demographic data is being collected, whilst balancing against proportionality, privacy and participant choice.  • Include the option of ‘prefer not to answer’ for all demographic questions.  • Utilise [Diversity and Inclusion Survey (DAISY) questions](https://edisgroup.org/resources/practical-tools-and-guidance/diversity-and-inclusion-survey-daisy-question-guidance-v2/) as part of diversity monitoring in research.  • Collect anonymised demographic data on eligible population who refuse participation or are not being approached.  • Collect data on reasons for participant drop out.  • Allow for proxy completion of data where possible (family members, friends, care workers or clinicians). |
| 25 | Report results stratified by under-represented characteristics or sub-groups. | • Describe study outcomes by under-represented characteristics.  • Consider conducting sub-group analysis by under-represented characteristics (pre-specify analysis by under-represented characteristics in the statistical analysis plan) |
|  | Impact, dissemination and engagement | |
| 26 | Document dissemination of research results to participants and wider relevant communities. | • Utilise mailing lists, social media platforms e.g. X (Twitter), and/or study-specific website to provide regular updates to participants.  • Upon study completion, confirm whether the participant would like to receive a copy of the study result and provide an approximate time frame as to when it may become available.  • Create easy-read flyers or short educational videos of the research results in relevant languages and disseminate widely to research participants, relevant patient population, communities, charity groups and more.  • Hold a ‘celebration event’ to share the research results and thank the wider patient and public contributions, including communities which have provided input into the research process. |

## Case Studies

The following case studies provide select examples of inclusive practices in cardiovascular research.

**Case study 1:** NIHR Leicester BRC Dance & Health Project, an innovative way of community engagement within existing research infrastructure

**Case study 2:** Deep End Research Alliance (DERA) and a model of community engagement through Community Research Link Workers (CRLWs)

**Case study 3:** Research Inclusion Plan from the CORAL study

**Case study 4:** BCIS-4, a multi-centre RCT using a hub and spoke delivery model

**Case study 5:** ROMA:WOMEN, an international RCT dedicated to women to improve coronary bypass outcomes

**Case study 6:** ADAPTABLE, an example of culturally tailored research recruitment materials

**Case study 7:** Using Artificial Intelligence (AI)-enabled pathway to increase participation of people who don’t speak English into research studies

### Case Study 1: NIHR Leicester BRC Dance & Health Project, an innovative way of community engagement within existing research infrastructure

**Summary**

The Dance and Health Project was established by the National Institute for Health Research (NIHR) Leicester Biomedical Research Centre (BRC) to promote public and community involvement in health research. It uses an innovative method of engagement – a weekly 60-min dance class to promote physical health, followed by 30 minutes of researcher-led discussion.

The Dance and Health Project was designed to reach participants from low-socioeconomic backgrounds and ethnic minority groups who are especially under-represented in cardiovascular research. Researchers can discuss research with communities in a safe space and fun environment.

The project’s commitment to inclusivity and cultural relevance has received overwhelming positive feedback from both community members and researchers and is an exemplar of effective community engagement in research. Community engagement facilitated through a pre-existing programme means that researchers do not necessarily need to establish new partnerships, which may be time and resource intensive.

**Key points**

- Innovative and creative methods can promote research engagement with under-represented communities.
- Community engagement facilitated through pre-existing partnerships where these are available saves time and money and should be used whenever possible.
- Community engagement needs to be long-term, instead of a tokenistic one-off activity.

***Additional information***

***- Website***	<https://leicesterbrc.nihr.ac.uk/get-involved/>

***- Publication***	Pritchard, R., Darko, N. & Stevenson, E. Enhancing community engagement, public involvement, and social capital through researchers’ participation in community dance projects: unexpected outcomes in ` communities. Res Involv Engagem **10**, 79 (2024). <https://doi.org/10.1186/s40900-024-00616-9>

***- Contact detail***	[NIHRLeicesterPPIE@uhl-tr.nhs.uk](mailto:NIHRLeicesterPPIE@uhl-tr.nhs.uk)

### Case Study 2: Deep End Research Alliance (DERA) and a model of community engagement through Community Research Link Workers (CRLWs)

*Written by Dr Kate Fryer, Co-Lead and Project Manager of DERA*

**Summary**

The Deep End Research Alliance (DERA) is a three-way partnership of patients, clinicians and academics. DERA aims to tackle health inequalities by designing and carrying out research in partnership with under-served communities, working at the intersection of ethnicity and socio-economic deprivation. Since 2021, DERA has been building partnerships with local communities and developed a model of community engagement that centres around Community Research Link Workers (CRLWs). The model works as follows:

1.	Identify key people and organisations representing local ethnic minority communities.

2.	Build relationships and establish trust.

3.	Identify and train CRLWs: generic and project-specific.

4.	Support CRLWs throughout study.

6.	Evaluate and co-produce next steps.

7.	Share outcomes of the study with the community.

We have shown that the CRLW model of engagement increases participation of people from ethnic minority communities in research when compared with other methods of engagement we have used in DERA. Community leaders and CRLWs said that sharing resources and power in the research process builds trust and interest in research participation in their communities.

DERA recommends a CRLW model which builds capacity and embeds reflective practice, mutual respect, and power sharing across the research team.

**Key points**

- Health research is often ‘exclusive by design’.
- CRLWs are critical in ensuring culturally appropriate research while providing a balanced voice to research projects.
- CRLWs should be involved across all stages of research, from priority setting to disseminating outputs.

***Additional information***

***- Website***	<https://sites.google.com/sheffield.ac.uk/dera/research-within-communities/community-researcher-toolkit?authuser=0>

***- Contact detail***	[DERA@Sheffield.ac.uk](mailto:DERA@Sheffield.ac.uk)

### Case Study 3: Example of Research Inclusion Plan from the CORAL study (COenzyme Q10 in heaRt fAiLure with reduced ejection fraction (CORAL, NIHR152257)

**Summary**

The CORAL study is a pragmatic randomised controlled trial (RCT) testing the effectiveness of co-enzyme Q10 in patients with heart failure. Participants are being recruited from General Practices across England. The CORAL researchers have developed and embedded an inclusivity intervention to increase participation of people from under-represented groups.

Intervention components include:

- targeted site selection in socioeconomically deprived areas
- recruitment and employment of Community Champions to engage minority populations
- cultural competency training for recruiting staff
- co-produced easy-read study materials
- interpreting and translation services, and
- financial incentives for all participants

**Key points**

- The inclusivity intervention was embedded and budgeted for at the design stage of the study.
- We will conduct an evaluation to collect data on the use, acceptability and cost of each intervention component.
- This evaluation will provide data to guide decisions about which inclusivity intervention components work best and should be incorporated in future studies.

**Additional information**

***- Website***	<https://coral-study.bristol.ac.uk/>

***- ISRCTN Registration***	<https://doi.org/10.1186/ISRCTN26946710>

**- Contact detail**	Dr Barbara Warnes, CORAL Trial Manager, [coral-study@bristol.ac.uk](mailto:coral-study@bristol.ac.uk)

Dr Rachel Johnson, CORAL co-Chief Investigator

Dr Maria Pufulete, CORAL co-Chief Investigator, [maria.pufulete@bristol.ac.uk](mailto:maria.pufulete@bristol.ac.uk)

### Case Study 4: BCIS-4, a multi-centre RCT using a hub and spoke delivery model

**Summary**

The BCIS4 trial compares the effectiveness of percutaneous coronary intervention (PCI, cardiac stents) and coronary artery bypass grafting (CABG, bypass surgery) for people with heart failure. It uses a hub and spoke study delivery model.

The hub and spoke model already exists in clinical care delivery and pathways – District General Hospitals (DGHs; spoke) offer angiography and PCI services, and may refer patients onwards to a regional tertiary hospital which offers CABG (hub). By utilising a hub and spoke model, more participants can be identified from geographically wider areas whilst reducing the need to travel long distances for research procedures. Similarly, smaller district hospitals are involved in research that would otherwise be delivered only in tertiary centres.

In BCIS4, participants may be identified at a PCI centre (site 1; spoke). If they were randomised to CABG, participants would attend the tertiary hospital for surgery (site 2; hub) and all research activities until the time of discharge would be conducted at the tertiary hospital. Subsequent research follow-up, however, would return to site 1 which is geographically closer to the participant’s home location. See image below for schematic summary.

There are logistical and research governance complexities which needs to be managed often on a bespoke basis. As an example, in order to provide informed consent, eligible participants at the spoke site would need to have the opportunity to discuss the risks and benefits with a surgeon at the hub site. If there is no pre-existing agreement at the service level, additional hurdles including financial agreements and research capacity will all need to be addressed.

The HRA provides a [template Hub and Spoke Agreements](https://www.myresearchproject.org.uk/help/hlptemplatesfor.aspx#HubSpokeAgreements) for consideration.

**Key points**

- The hub and spoke model includes more local hospitals that may not otherwise participate in research.
- This may yield additional eligible participants as well as improved retention rate.
- This model of delivery can be complex and participant pathways need to be carefully considered along with research governance and logistical challenges.

***Additional information***

***- ISRCTN Registration***	<https://doi.org/10.1186/ISRCTN29654606>

***- Contact detail***	[bcis-4@leicester.ac.uk](mailto:Bcis-4@leicester.ac.uk)

### Case Study 5: ROMA:WOMEN, an international RCT dedicated to women to improve coronary bypass outcomes

**Summary**

ROMA:Women is the first cardiac surgery trial enrolling only women. This approach is necessary as women are under-represented in cardiovascular research and specifically in research on the use of multiple arterial grafting (MAG) in coronary artery bypass grafting (CABG). Enrolling only women in the ROMA:Women trial addresses this evidence gap and ensures an adequate sample size to definitively answer whether MAG is best for women CABG patients.

There are several strategies which have proved successful in trial design and increasing recruitment of women:

1. Having women in the majority of Steering Committee positions (>75%)
2. Having patient representatives on the Steering Committee and participating in oversight
3. Strongly encouraging identification of women as principal investigators and study personnel
4. Interviewing women who declined to participate in ROMA or ROMA:Women, to obtain qualitative perspectives on why they chose not to participate
5. Nested trial design (or RCT within RCT). This makes use of the existing ROMA trial infrastructure and allows incorporation of all women patients from the main ROMA trial (n=690)
6. Actively promoting the ROMA:Women trial in the cardiovascular community through presentation at meetings, newsletters, social media, and email outreach.

UK enrolment is currently ahead of target and this approach is an example for cardiovascular trialists to consider when designing trials for women or other under-represented groups.

**Key points**

- Women are under-represented in cardiac surgery trials.
- ROMA:Women is the first cardiac surgery trial dedicated to women and will inform sex-specific CABG guidelines regarding the use of multiple arterial grafts in women.
- Designing trials specific for under-represented groups is feasible and can be used to address evidence gaps.

***Additional information***

***- Website***	<https://www.theromatrialwomen.com/>

***- Publications***	Gaudino M, Bairey Merz CN, Sandner S, et al. Randomized Comparison of the Outcome of Single Versus Multiple Arterial Grafts trial (ROMA):Women-a trial dedicated to women to improve coronary bypass outcomes. *J Thorac Cardiovasc Surg*. 2024;167(4):1316-1321. doi:10.1016/j.jtcvs.2023.06.006

Gaudino M, Fremes SE, Mehran R, Bairey Merz CN; ROMA:Women Steering Committee and Investigators. ROMA:Women: Innovative Approaches for the First Cardiac Surgery Trial in Women. *Circulation*. 2023;148(17):1289-1291. doi:10.1161/CIRCULATIONAHA.123.064033

***- Contact detail***	roma@le.ac.uk

### **Case Study 6**: ADAPTABLE, an example of culturally tailored research recruitment materials

**Summary**

Cultural tailoring of recruitment materials is often suggested as a strategy to promote the enrolment of under-represented groups in clinical trials. However, there is a lack of guidance for research teams to create such materials. In this study, the authors described the development of guidelines for cultural tailoring of recruitment material for African Americans and Latinos. The developed guideline was pilot tested in the ADAPTABLE study, a large pragmatic trial on the optimal dose of aspirin for secondary prevention in patients with atherosclerotic cardiovascular disease.

Specific considerations for images and language used in recruitment material were suggested depending on the population of interest:

- Use of visuals recognised by the population (e.g. image of research participants)
- Use of preferred terminology by the population (e.g. avoid using ‘disease’ which has a negative connotation, or use inviting words such as ‘learn’ instead of ‘join’)
- Address informational needs specific to the population (e.g. emphasis on confidentiality)
- Specify incentives for study participation (e.g. health risks and benefits)

Tailored vs non-tailored recruitment banner for Facebook campaign in the ADAPTABLE study is presented below. In the pilot study, the authors reported significantly higher clicks per impression ratio and enrolled a greater percentage of African American using tailored compared to non-tailored ads (13% vs 8%, respectively).

Table 1 above is reproduced without changes from Cunningham-Erves et al [1] under [Creative Commons Attribution 4.0 International License](http://creativecommons.org/licenses/by/4.0/).

**Key points**

- Cultural tailoring of recruitment material can and should be done, starting with a focus on language and images.
- Participant facing materials, including recruitment material and participant information leaflet, should be co-produced with the population of interest.

***Additional Information***

***- Publication***	[1] Cunningham-Erves, J., Kusnoor, S.V., Villalta-Gil, V. *et al.* Development and pilot implementation of guidelines for culturally tailored research recruitment materials for African Americans and Latinos. *BMC Med Res Methodol* **22**, 248 (2022). https://doi.org/10.1186/s12874-022-01724-4

### Case Study 7: Using Artificial Intelligence (AI)-enabled pathways to increase participation of people who don’t speak English into research studies

**Summary**

People who do not speak English well or at all are often excluded from research studies because it can be difficult to organise an interpreter and translate patient information material quickly enough to introduce the study in their language.

For example, people who have urgent heart surgery (about half of the heart surgery population) are transferred from their local hospital to the heart surgery centre at short notice and often on the day before their operation. Research nurses therefore only have a narrow window in which to introduce a research study to them.

We designed an AI translation pathway making use of a hand-held device called the Pocketalk to translate the first conversations that researchers have with people about a study and online translators to rapidly translate the patient information leaflet.

In preliminary testing within our team, we found that simple and concise English language avoiding jargon and colloquialisms, short sentences, subject-verb-object sentence structure, using the same verb tenses throughout resulted in good translations in all languages tested (Romanian, Italian, Portuguese, Spanish, Arabic, German, Malayalam)

We have applied for funding (NIHR research for Patient Benefit) to conduct a proof-of-principle study to test this AI translation pathway.

**Key points**

- Using online translators allows recruiting staff to introduce a study to a potential participant quickly in their language.
- This can be followed up with standard NHS interpreting / translation service.
- We are conducting a study to test the usability and acceptability of the AI translation pathway with patients and recruiters.

**Additional information**

***- Website***	[*https://www.pocketalk.com/en_gb/*](https://www.pocketalk.com/en_gb/)

***- Contact***	Emma Hopkins (Cardiac Research Sister, Bristol Heart Institute): [emma.hopkins@uhbw.nhs.uk](mailto:emma.hopkins@uhbw.nhs.uk)

Maria Pufulete (Bristol Medical School): [maria.pufulete@bristol.ac.uk](mailto:maria.pufulete@bristol.ac.uk)

## Appendices

The following appendices provide additional resources; however, they are not intended to be exhaustive. If you are aware of additional resources and would like them added to the appendices please contact [heartsurgerypsp@leicester.ac.uk](mailto:heartsurgerypsp@leicester.ac.uk).

**Appendix 1.** Existing PPI Groups in Cardiovascular Research

**Appendix 2.** Examples of terms of Reference for Patient and Public Involvement; Job specification for Community Researchers

**Appendix 3.** Template for costing research inclusion

**Appendix 4.** External vendors for research recruitment

**Appendix 5.** Additional resources to consider when creating participant-facing material

### Appendix 1. Existing PPI Groups in Cardiovascular Research

| **Group** | **Brief Description** | **Contact** |
| --- | --- | --- |
| National Cardiac Surgery PPI Group | Established as part of the National Cardiac Surgery Cardiac Trials Initiative, the National Cardiac PPI Group is a collection of committed individuals who have lived experience of heart disease in one form or another. They work alongside clinicians and researchers to develop research trials that mean something to patients. | <https://le.ac.uk/cardiovascular-sciences/about/heart-surgery/national-ppi-group>  Email: [heartsurgerypsp@leicester.ac.uk](mailto:heartsurgerypsp@leicester.ac.uk)  Phone*:* +44(0)116 252 2188 |
| Heart Research UK Patient and Public Network | Patient and public members living in the UK with experience of living with or caring for someone with a heart condition. The group’s purpose is to help form HRUK’s strategy, inform and guide their charity activities and the research they fund. | <https://heartresearch.org.uk/patient-and-public-network/>  Email: [info@heartresearch.org.uk](mailto:info@heartresearch.org.uk)  Phone: +44(0) 113 234 7474 |
| BHF Heart Voices | Community of people with lived experience with a heart or circulatory condition who help shape the work at British Heart Foundation. | <https://www.bhf.org.uk/for-professionals/information-for-researchers/how-to-apply/patient-and-public-involvement>  Email: [heartvoices@bhf.org.uk](mailto:heartvoices@bhf.org.uk) |
| Heart Valve Voices | UK’s dedicated heart valve disease charity who works with patients and clinicians to help increase awareness of heart valve disease. | <https://heartvalvevoice.com/>  Email: [info@heartvalvevoice.com](mailto:info@heartvalvevoice.com) |
| The Aortic Dissection Charitable Trust | UK & Ireland charity uniting patients, families and the medical community in a shared goal of improving diagnosis, increasing survival and reducing disability due to aortic dissection. | <https://aorticdissectioncharitabletrust.org/>  Email: [heretohelp@tadct.org](mailto:heretohelp@tadct.org) |
| Take Heart Leicester | Local patient support group for adult cardiac patients, based at the University Hospitals of Leicester. | <https://takeheartleicester.co.uk/>  Email: [thl@takeheartleicester.co.uk](mailto:thl@takeheartleicester.co.uk)  Phone: +44(0) 7970075039 |
| Cardiovascular Care Partnership UK (CCPUK) | National cardiovascular charity, affiliated to the British Cardiovascular Society (BCS) which supports patients with cardiovascular disease and associated conditions, their carers and also other cardiovascular support groups.  The group’s overall purpose is to be the patient & carer voice for the BCS. | <https://www.ccpuk.org.uk/>  Email: [ccp@bcs.com](mailto:ccp@bcs.com) |
| International Heart Spasms Alliance | International Heart Spasms Alliance (IHSA) is dedicated to supporting those affected by unseen heart issues.  IHSA serves both the patient and medical communities by educating, informing, and enlightening them to help achieve earlier diagnoses, better treatments, and support research into these conditions. | <https://www.internationalheartspasmsalliance.org/>  Email: [ihsacontact@gmail.com](mailto:ihsacontact@gmail.com) |
| UK Vascular Research PPI Registry | A list of volunteers with personal experience of, or an interest in, vascular conditions such as PAD and Abdominal Aortic Aneurysm (AAA). Members on the Registry are contacted when new vascular research studies are being discussed by health professionals, to ensure that patient and public opinions and experience are included within the design. | Email: [PPIVasc@leicester.ac.uk](mailto:PPIVasc@leicester.ac.uk) |
| Pumping Marvellous | The UK’s patient led heart failure charity. The Foundation’s goal is to deliver HOPE to its recipients through the facilitation of better outcomes by cross-working and advocating at a local, regional, national, and international level. Working hand-in-hand with all stakeholders to deliver better pathways and be the patient voice of progression. | <https://pumpingmarvellous.org/>  Email: [hearts@pumpingmarvellous.org](mailto:hearts@pumpingmarvellous.org)  Phone: +44(0) 177 279 6542 |
| Somerville Heart Foundation | The only UK-wide charity dedicated to supporting young people and adults born with all forms of Congenital Heart Disease.  They provide support, advocacy, communication, and a community for those we are here to help. They also campaign for their rights to receive excellent medical care to enable them to lead happy, healthy, and longer lives. | <https://sfhearts.org.uk/>  Email: [info@sfhearts.org.uk](mailto:info@sfhearts.org.uk)  Phone: +44(0) 1473 252007 |

### Appendix 2. Examples of Terms of Reference for Patient and Public Involvement; and Job Specification for Community Researchers

The roles of patient, community, or public members as research partners need to be clearly defined. Below we provide the Terms of Reference of a National PPI group and an exemplar job specification for a patient as researcher, which can be applied to the role of a community researcher.

- [National Cardiac Surgery PPI Group Terms of Reference](https://drive.google.com/file/d/1oLZ26oZVWjgcKu8VYlXlHpEh2vyCXh87/view?usp=sharing)
- [Job specification for a patient researcher](https://drive.google.com/file/d/1HaQHywg0Ss57pRCVazbRWYqY48SWyEpd/view?usp=sharing), which can be applied to community researcher.

### Appendix 3. Costing for research inclusion

No standardised costing template exists for costing Research Inclusion. The NIHR, which mandates an inclusive research design as a condition of funding, provides the following advice:

[NIHR Finance guidance for applicants](https://www.nihr.ac.uk/research-funding/application-support/guidance/finance-guidance-for-applicants#tab-375176), see “Research inclusion” under Costing assumptions

Include any costs related to ensuring your research is designed, recruited, delivered, and mobilised inclusively. This may be (but is not limited to) costs associated with:

- upskilling research teams with additional expertise relevant to equality, diversity and inclusion. For example, training in understanding how to work with specific groups relevant to the research, health inequalities, reasonable adjustments etc.
- inclusive recruitment materials or strategies to attract and retain diverse participants, or those from under-served communities and places
- adjustments to research materials, protocols, methods, or events. This ensures participation and retention of diverse groups
- purchasing specialist software or services
- additional or alternative data collection. This is to ensure data collected from the relevant population is robust, or that findings can be fully applied to different populations
- additional sites or locations to carry out your research
- inclusively mobilising research findings

Clearly detail staff costs associated with research inclusion should be clearly indicated and included in the ‘Staff costs’ section. NIHR acknowledges that research delivered inclusively may take longer, and this may result in increased staff costs.

Costings for reasonable adjustments that you, or any co-applicants may require, should not be included under the research inclusion costing. Please attribute these costs under ‘Other direct costs’.

The following items may be helpful to consider when costing for research inclusion. They are by no means exhaustive, and the exact items will depend on the study proposed and its Research Inclusion Plan.

**Budget Item**

**Staff cost**

Community researcher(s)

Community Engagement Officers

Research Inclusion lead

**Patient, public, and community involvement**

Community engagement (consider venue, travel, catering, access to facilities, and any consumables)

PPI contributor payment if members of the community are part of study / trial steering group (consider the number of meetings involved throughout the whole research process)

Donation or financial support for community / patient group

**Direct costs in research design and delivery**

Non-conventional trial design (costing will be considered in SoECAT)

Advertisement cost (e.g. online and offline, printing, advert fees)

External vendor for recruitment assistance

Participant incentives

Financial reimbursement (consider food, travel, accommodation, childcare, carer costs)

Staff travel to participants

**Communication & accessibility cost**

Staff training on research inclusion

Development of communication resources (visual aids, videos, and/or animations)

Translation and back-translation costs for all recruitment material

Interpreter cost

Braille and audio material

Assistive technology hire

Any other reasonable adjustments required for research participation (e.g. increased visit time)

Participant testimonial videos

Administrative costs (e.g. study-specific website, telephone costs, postal stamps, and stationary)

**Dissemination**

Outreach and education events (consider venue, travel, catering, access to facilities, and any consumables) to disseminate result to relevant communities

Celebration event

### Appendix 4. External vendors for research recruitment.

| **Group** | **Website** |
| --- | --- |
| COUCH Health | <https://couch.health/> |
| Egality Health | <https://egality.health/> |
| Health Watch | <https://www.healthwatch.co.uk/> |
| Caafi Health | <https://www.caafihealth.org.uk/> |

* Disclaimer: the authors of this checklist have no experience or affiliation with external vendors for inclusive research recruitment.

### Appendix 5. Additional resources to consider when creating participant-facing material

| **Resource** | **Website** | **Description** |
| --- | --- | --- |
| Guidance on creating and designing recruitment materials | | |
| NHS Health Research Authority (HRA) | <https://www.hra-decisiontools.org.uk/consent/examples.html>  <https://www.hra.nhs.uk/planning-and-improving-research/research-planning/participant-information-design-and-review-principles/> | Examples and templates of participant information sheets from the Health Research Authority’s Consent and Participant Information Guidance |
| NIHR Good Practice Guide: Inclusive Language and Communications | <https://learn.nihr.ac.uk/course/view.php?id=1181>  (NIHR login required) | This outlines and provides context to the five key principles for NIHR affiliated researchers to communicate inclusively. |
| University of Florida, Clinical and Translational Science Institute | <https://www.ctsi.ufl.edu/research/participant-recruitment/recruitment-flyers/best-practices-for-recruitment-flyers/> | Example patient information sheet with highlighted best practices on creating participant facing recruitment materials |
| University of North Carolina, Translational and Clinical Sciences Institute | <https://tracs.unc.edu/docs/recruitment/Recruitment_Designing_Effective_Recruitment_Materials_20211006.pdf> | Training presentation on designing effective participant facing recruitment materials |
| The Plain English Campaign | <https://www.plainenglish.co.uk/free-guides.html> | Series of guides to aid the use of plain English in written materials. |
| Readability checkers | | |
| Flesch-Kincaid Grade Level test and Flesch Reading Ease test within Microsoft Office | <https://support.microsoft.com/en-gb/office/get-your-document-s-readability-and-level-statistics-85b4969e-e80a-4777-8dd3-f7fc3c8b3fd2> | A function which is available within Microsoft Words. Free online calculators also available from web searches. |
| Gunning Fog Index | <http://gunning-fog-index.com> | A readability checker that generates an index score by reading level. |
| Hemmingway Editor | <https://hemingwayapp.com/> | Hemmingway text editor, including readability checker. |
| Creating accessible documents | | |
| Leeds Beckett University | <https://teachlearn.leedsbeckett.ac.uk/-/media/files/clt/guide-to-alternative-and-accessible-formats.pdf> | Leeds Beckett guide on providing materials in accessible and alternative formats. |
| Accessibility checker within Microsoft Office | <https://support.microsoft.com/en-gb/office/improve-accessibility-with-the-accessibility-checker-a16f6de0-2f39-4a2b-8bd8-5ad801426c7f#PickTab=Windows> | Guidance on using in-built accessibility checker, currently only available on Microsoft Word but soon to be available for other office desktop apps. |

## Funding

This research was funded by the following grants; Heart Research UK TDG23-100002, NIHR150600, NIHR155123, BHF CS/F/23/190055, and the Leicester NIHR Biomedical Research Centre. AC is supported by NIHR304685 and BHF AA/18/3/34220. GJM and HA are supported by the British Heart Foundation (CH/12/1/29419). SM is supported by NIHR150600. SP is supported by HRUK TDG23-100002.

## Core development team

| **Dr Ann Cheng**  Clinical Research Fellow  University of Leicester | **Dr Maria Pufulete**  Associate Professor in Applied Health Research  University of Bristol | **Prof Gavin Murphy**  British Heart Foundation Chair of Cardiac Surgery  University of Leicester |
| --- | --- | --- |
| **Mr Hardeep Aujla**  Senior Research Manager  University of Leicester | **Dr Robert Grant**  Clinical Research Fellow  University of Leicester | **Mrs Sue Page**  Project Manager  National Cardiac Surgery Clinical Trials Initiative |
| **Mrs Sarah Murray** Chair  National Cardiac Surgery PPI Group | **Mr Jeremy Dearling**  Patient and public representative  National Cardiac Surgery PPI Group |  |

## Contributors

**National Cardiac Surgery PPI Group**

**Community members** from the NIHR Leicester BRC Dance & Health Project

**Mrs Carol Akroyd**

Associate Director

ARC East Midlands

**Dr Ali Alhashimi**

Foundation Year Doctor

Sheffield Teaching Hospitals NHS Trust

**Dr Maria Gonzalez Aguado**

Qualitative Research Associate

University of Leicester

**Prof Enoch Akouwah**

Academic Consultant Cardiothoracic Surgeon

South Tees NHS Foundation Trust

**Ms Allyson Arnold**

Chief Operating Officer

British Heart Foundation Clinical Research Collaborative

**Mrs Marta Barbieri**

Patient and public representative

**Prof Julian Barwell**

Professor in Genomic Medicine

University of Leicester

**Mr David Batchelor**

Patient and public representative

National Cardiac Surgery PPI Group

**Ms Joanna Bell**

Public Engagement Coordinator

NIHR Leicester Biomedical Research Centre

**Prof Farah Bhatti, OBE**

Consultant Cardiothoracic Surgeon

Swansea University

**Mrs Jayshree Bhjikha**

Patient and public representative

**Ms Judith Bower**

Patient and public representative

National Cardiac Surgery PPI Group

**Ms Anjie Chhapia**

Patient and public representative

**Dr Lucy Culliford**

Associate Professor in Clinical Trial Methodology

University of Bristol

**Ms Samantha Dias**

Patient and public representative

**Dr Kate Fryer**

Research Fellow

University of Sheffield

**Mr Ray Gandhi**

Patient and public representative

**Ms Hannah Gilbert**

Trials Manager Leicester CTU (past)

University of Leicester

**Dr Laura Green**

Associate Professor of Haematology

Queen Mary University of London

**Ms Anab Hasan**

PhD Candidate

University of Leicester

**Dr Noreen Hopewell-Kelly**

Patient and public representative

Cardiff University

**Ms Emma Hopkins**

NIHR Senior Research Leader and Cardiac Surgery Research Sister

Bristol Heart Institute

**Ms Lathishia Joel-David**

Senior Research Nurse

Glenfield Hospital

**Dr Christina King**

Patient and public representative

**Prof Gudrun Kunst**

Professor of Cardiovascular Anaesthesia

King’s College London

**Ms Janet Kyte**

Patient and public representative

**Mr Mark Lewis**

Patient and public representative

National Cardiac Surgery PPI Group

**Mr Anthony Locke**

Patient and public representative

National Cardiac Surgery PPI Group

**Prof Mahmoud Loubani**

Consultant Cardiac Surgeon

Hull University Teaching Hospitals NHS Trust

**Ms Rosalie Magboo**

Matron and Clinical Doctoral Research Fellow

St Bartholomew’s Hospital

Queen Mary University of London

**Dr Rebecca Maier**

Head of South Tees Academic Cardiovascular Unit

South Tees NHS

**Mr Harish Mistry**

Patient and public representative

**Dr Chibuzo Mowete**

NHSBT Clinical Research Fellow

Queen Mary University of London

**Mr Ayodeji Ogunbuyide**

Community Engagement Manager

NIHR Leicester Biomedical Research Centre

**Mr Gareth Owens**

Past chair

Aortic Dissection Awareness UK and Northern Ireland

**Mrs Sushila Pankhania**

Patient and public representative

**Ms Jess Plant**

Patient and public representative

**Dr Alison Prowle**

Senior Lecturer

University of Worcester

**Dr Muhammad Rashid**

NIHR DSE Senior Clinical Research Fellow

University of Leicester

**Prof Julie Sanders**

Professor of Cardiovascular Care

King’s College London

**Prof Athanasios Saratzis**

Professor of Vascular Surgery

University of Leicester

**Dr Anvesha Singh**

Associate Professor and Consultant Cardiologist

University of Leicester

**Mrs Sally Spawforth**

Patient and Public Involvement Officer

Heart Research UK

**Mr Gil Wheeler**

Patient and public representative

National Cardiac Surgery PPI Group

**Ms Cathy Wield**

Patient and public representative

**Mr Keith Wilson**

Patient and public representative

National Cardiac Surgery PPI Group

Liverpool Heart and Chest Hospital

## References

1. Collins SE, Clifasefi SL, Stanton J, The Leap Advisory Board, Straits KJE, Gil-Kashiwabara E, Rodriguez Espinosa P, Nicasio AV, Andrasik MP, Hawes SM, Miller KA, Nelson LA, Orfaly VE, Duran BM, Wallerstein N. Community-based participatory research (CBPR): Towards equitable involvement of community in psychology research. Am Psychol. 2018 Oct;73(7):884-898. doi: 10.1037/amp0000167. Epub 2018 Jan 22. PMID: 29355352; PMCID: PMC6054913.
2. Holkup PA, Tripp-Reimer T, Salois EM, Weinert C. Community-based participatory research: an approach to intervention research with a Native American community. ANS Adv Nurs Sci. 2004 Jul-Sep;27(3):162-75. doi: 10.1097/00012272-200407000-00002. PMID: 15455579; PMCID: PMC2774214.
